# Targeting AMP‐activated kinase impacts hepatocellular cancer stem cells induced by long‐term treatment with sorafenib

**DOI:** 10.1002/1878-0261.12488

**Published:** 2019-04-15

**Authors:** Alicia Bort, Belén G. Sánchez, Pedro A. Mateos‐Gómez, Diana Vara‐Ciruelos, Nieves Rodríguez‐Henche, Inés Díaz‐Laviada

**Affiliations:** ^1^ Department of Systems Biology School of Medicine University of Alcala Alcalá de Henares, Madrid Spain; ^2^ Division of Cell Signalling & Immunology College of Life Sciences University of Dundee UK; ^3^ Chemical Research Institute ‘Andrés M. del Río’ (IQAR) Alcalá University Alcalá de Henares, Madrid Spain

**Keywords:** AMP‐activated kinase, cancer stem cells, drug resistance, hepatocellular carcinoma, sorafenib

## Abstract

Hepatocellular carcinoma (HCC) is the third leading cause of cancer death worldwide. HCC treatment is hindered by the frequent emergence of chemoresistance to the multikinase inhibitor sorafenib, which has been related to the presence of cancer stem cells (CSCs) that self‐renew and often escape therapy. The key metabolic sensor AMP‐activated kinase (AMPK) has recently been recognized as a tumour growth regulator. In this study, we aimed to elucidate the role of AMPK in the development of a stem cell phenotype in HCC cells. To this end, we enriched the CSC population in HCC cell lines that showed increased expression of drug resistance (ALDH1A1, ABCB1A) and stem cell (CD133, Nanog, Oct4, alpha fetoprotein) markers and demonstrated their stemness phenotype. These cells were refractory to sorafenib‐induced cell death. We report that sorafenib‐resistant cells had lower levels of total and phosphorylated AMPK as well as its downstream substrate, ACC, compared with the parental cells. Interestingly, AMPK knockdown with siRNA or inhibition with dorsomorphin increased the expression of stem cell markers in parental cells and blocked sorafenib‐induced cell death. Conversely, the upregulation of AMPK, either by transfection or by pharmacological activation with A‐769662, decreased the expression of ALDH1A1, ABCB1A, CD133, Nanog, Oct4, and alpha fetoprotein, and restored sensitivity to sorafenib. Analysis of the underlying mechanism points to hypoxia‐inducible factor HIF‐1α as a regulator of stemness. *In vivo* studies in a xenograft mouse model demonstrated that stem‐like cells have greater tumourigenic capacity. AMPK activation reduced xenograft tumour growth and decreased the expression of stem cell markers. Taken together, these results indicate that AMPK may serve as a novel target to overcome chemoresistance in HCC.

**Abbreviations**
ACC: acetyl‐CoA carboxylase
AFP: alpha fetoprotein
ALDH1A1: aldehyde dehydrogenase isoform 1A1
AMP: adenosine monophosphate
AMPK: AMP‐activated kinase
CAP: capsaicin
CSC: cancer stem cell
GFAP: glial fibrillary acidic protein
HCC: hepatocellular carcinoma
HIF‐1α: hypoxia‐inducible factor 1α
Oct4: octamer‐binding transcription factor 4
PGC1α: proliferator‐activated receptor‐γ coactivator 1‐α
PPARγ: proliferator‐activated receptor‐γ
SF: sorafenib

## 1 Introduction

Cancer continues to be the second leading cause of death in Western countries. More than 2 million new cases of cancer and 610 000 cancer deaths are predicted to occur only in the United States during 2018 (Siegel *et al*., 2018). Hepatocellular carcinoma (HCC) is the most common liver malignancy in adults and the third leading cause of cancer mortality worldwide (Beal *et al*., 2017). The prognosis for patients with advanced HCC remains extremely poor due to the high resistance to cytotoxic therapy. For the past decade, the multikinase inhibitor sorafenib has been the only approved standard treatment for patients with advanced HCC (Kudo, 2017), albeit it only extends life expectancy by 2–3 months (Cheng *et al*., 2009; Llovet *et al*., 2008; Tovoli *et al*., 2018). This can be attributed to the acquisition of resistance, predominantly due to the upregulation of certain survival pathways that may cover up the death signals induced by sorafenib (Zhu *et al*., 2017). Several clinical trials have investigated other multitargeted kinase inhibitors, but none have shown any benefits over single‐agent sorafenib. In August 2018, the Food and Drug Administration approved lenvatinib for first‐line treatment of patients with unresectable hepatocellular carcinoma based on overall survival results obtained in clinical trials (Kudo, 2018; Spallanzani *et al*., 2018). Nevertheless, it is still too early to know whether this drug produces long‐term secondary effects or resistance. Hence, the elucidation of the underlying mechanisms of evasive resistance is required to overcome unwanted tumour recurrence and, consequently, to improve the beneficial effects of chemotherapy.

Drug resistance to conventional chemotherapeutic agents has been closely related to many intrinsic or acquired properties associated with the presence of cancer stem cells (CSCs), which possess higher proliferative output and the ability to self‐renew. CSCs overexpress drug resistance genes, such as drug efflux transporters or detoxifying enzymes, which confer protection from the adverse effects of chemotherapeutic insult (Begicevic and Falasca, 2017). Therefore, to prevent drug resistance and tumour recurrence, it is imperative to gain a better understanding of the mechanisms involved in the resistance of stem cells to chemotherapy, which could lead to the discovery of new targets that can be exploited with a therapeutic purpose.

Previous research has shown that the isolation of hepatocellular CSC (HCC‐CSC) can be performed using cell surface stemness‐associated markers, such as the transmembrane glycoprotein CD133, alpha fetoprotein (AFP), or aldehyde dehydrogenase isoform 1A1 (ALDH1A1), which have been used for further HCC‐CSC classification into different prognostic subtypes (Dai *et al*., 2018; Ma *et al*., 2008a). In particular, HCC‐CSCs expressing CD133 were associated with a poor prognosis because they bear great tumourigenic potential, possess greater colony‐forming efficiency and display chemoresistance to the classical anticancer drugs doxorubicin and fluorouracil (Ma *et al*., 2008b). Although the underlying molecular mechanisms of chemoresistance in these cells are not completely clear, the data indicate sustained activation of the PI3K/Akt cascade (Ma *et al*., 2008b). In line with this, signalling pathways involved in the acquired resistance to sorafenib include activation of the PI3K/Akt and JAK/STAT axes, the induction of HIF‐1α‐mediated adaptation to hypoxia or the upregulation of FGF signalling pathways (Zhu *et al*., 2017). Similarly, recent findings showed that the sensitivity to sorafenib after developing resistance can be restored with PI3K/Akt inhibitors (Wu *et al*., 2016; Yi *et al*., 2017).

Our recent research on HCC resistance demonstrated that activation of the enzyme AMP‐activated kinase (AMPK) inhibits the PI3K/Akt pathway and sensitizes HCC cells to sorafenib (Bort *et al*., 2017). In addition, we demonstrated that the activation of AMPK by cannabinoids induces autophagy and suppresses HCC cell proliferation (Vara *et al*., 2011). Moreover, emerging evidence suggests that metformin, an AMPK activator used as an antidiabetic drug, resensitizes resistant cancer cells to chemotherapy by targeting several signalling pathways, including AMPK (Ling *et al*., 2014; You *et al*., 2016). In this regard, recent evidence demonstrates that metformin inhibits CSC proliferation (Finley, 2017; Saini and Yang, 2018) and reduces the expression of stemness markers (Paiva‐Oliveira *et al*., 2018). Therefore, AMPK emerges as a new target that could be involved in drug resistance and the development of CSCs.

In this study, we analysed the role of AMPK in the development of stem‐like cells and in the sorafenib resistance of HCC cells. We show that the downregulation of AMPK plays a key role in mediating an increase in the expression of stemness markers and that the pharmacological activation of AMPK or AMPK overexpression overcomes this stem cell‐like phenotype. Furthermore, sorafenib resistance was associated with stemness and was abrogated by AMPK transfection or activation. Our results expand the role of AMPK in cancer and highlight novel treatment options for resistant cancer.

## 2 Materials and methods

### 2.1 Reagents and antibodies

Sorafenib was purchased from Sigma‐Aldrich (St. Louis, MO, USA). The compound A‐769662 and capsaicin were purchased from Tocris Bioscience (Bristol, UK). The primary antibodies anti‐CD133, anti‐ALDH1A1, anti‐pAkt‐ser473, p‐mTOR, pAMPKα1‐thr172, pACC‐ser79, anti‐cyclin D1, anti‐PGC1α, and anti‐PPARγ and the antibodies against the corresponding total forms were obtained from Cell Signaling Technology (Danvers, MA, USA). The primary antibody anti‐β‐catenin was purchased from Santa Cruz Biotechnology (Dallas, Texas, USA). The primary antibody anti‐Hif‐1α was purchased from Novus (St. Louis, MO, USA). Anti‐alpha fetoprotein and peroxidase‐labelled secondary anti‐mouse IgG were purchased from Sigma‐Aldrich, and anti‐rabbit IgG was purchased from Calbiochem (San Diego, USA).

### 2.2 Cell lines and cell culture

The human hepatocellular carcinoma HepG2 cell line was purchased from the American Type Culture Collection (ATCC HB‐8065, Rockville, MD, USA). The human hepatoma cell line Huh7 was kindly provided by L. Boscá (Instituto de Investigaciones Biomédicas Alberto Sols, Madrid). Cell lines were incubated at 37 °C in a humidified atmosphere with 5% CO_2_ and cultured in DMEM/10% FBS supplemented with 1% nonessential amino acids, 100 IU·mL^−1^ penicillin G sodium, 100 μg·mL^−1^ streptomycin sulphate, and 0.25 μg·mL^−1^ amphotericin B (Invitrogen, Paisley, UK).

To generate cancer stem‐like cells, HepG2 and Huh7 cells were cultured continuously for 12 months with a step‐wise increase in the sorafenib concentration (starting at 0.75 μm and increasing the concentration by 0.15 μm at each passage up to a final concentration of 8 μm). Surviving cells were selected and designated as HepG2SF1 and Huh7SF1 cells. HepG2 and Huh7 parental cells were cultured in parallel without sorafenib and served as controls.

### 2.3 Cell proliferation assay

Cell proliferation was analysed using the MTT assay. Briefly, 5 × 10^3^ cells/well was seeded into 12‐well plates and allowed to attach and grow for 24 h. After treatment with sorafenib for 24 h, 200 μL of MTT (3‐(4,5‐dimethyl‐2‐thiazolyl)‐2,5‐diphenyl‐2H‐tetrazolium bromide) dye solution (Sigma‐Aldrich) was added to each well and incubated at 37 °C for 4 h. Subsequently, the cells were lysed with 2‐propanol to dissolve the formazan crystals. Then, the optical density of each well was measured using a microplate reader (iMARK, Bio‐Rad Laboratories, Inc., Hercules, CA, USA) at a wavelength of 595 nm, and the nonspecific absorbance measured at 650 nm was subtracted. Each experiment was performed in triplicate. Cell viability was calculated as the percentage compared to the control cells, which were arbitrarily assigned 100% viability. The half‐maximal inhibitory concentration (IC50) values, defined as the concentration that inhibited 50% cell growth relative to control cells, were calculated using graphpad 6.0 (La Jolla, CA, USA) software.

Cell viability was also determined by counting viable and dead cells by Trypan blue staining. Trypan blue‐positive and blue‐negative cells were counted using a Countess automated cell counter (Invitrogen, Carlsbad, CA, USA). The results are expressed in relation to the total number of cells counted.

### 2.4 Colony forming assay

Cells were seeded in flat‐bottom six‐well plates at different densities. Two weeks later, the medium was removed. Colonies were fixed in methanol, stained with 0.05% (w/v) crystal violet solution for 5 min, washed with PBS and counted.

### 2.5 Differentiation assay

For the *in vitro* differentiation of stem‐like cells into neurons, stem‐like cells (1 × 10^5^ cells/well) were seeded into 6‐well plates and incubated in phenol red‐free neurobasal medium (Invitrogen) supplemented with 2% B‐27 serum‐free supplement (Invitrogen), 2% CSS, and 2 mm l‐glutamine (Invitrogen) for 15 days. For glial redifferentiation, stem‐like cells were incubated in phenol red‐free DMEM (Sigma‐Aldrich) with 1% N‐2 supplement (Invitrogen), 2% CSS and 2 mm l‐glutamine (Invitrogen).

### 2.6 Western blot analysis

After treatment or transfection for 48 h, cells were harvested, and proteins were extracted using lysis buffer (50 mm Tris, pH 7.4, 0.8 m NaCl, 5 mm MgCl_2_, 0.1% Triton X‐100) containing protease inhibitor and phosphatase inhibitor cocktail (Roche, Diagnostics; Mannheim, Germany), incubated on ice for 15 min and cleared by microcentrifugation. Protein concentrations were measured using the Bio‐Rad™ protein assay kit (Richmond, CA, USA). The cell protein extracts (20 μg) were boiled for 5 min in loading buffer and then separated on 8–15% SDS/PAGE gels depending on the protein to be analysed. The separated protein bands were transferred onto a PVDF membrane and incubated with the primary antibodies diluted 1 : 1000 overnight at 4 °C. Horseradish peroxidase‐conjugated goat anti‐mouse and goat anti‐rabbit IgG secondary antibodies were then added at a dilution ratio of 1 : 2000, and the membranes were incubated at room temperature for 2 h. The immune complex was visualized with an ECL system (Cell Signaling Technology).

### 2.7 Flow cytometry

A total of 5 × 10^5^ HCC cells were seeded into 6‐well plates and treated according to the experiment. The cells were then harvested in 0.35% trypsin, collected and centrifuged at 1500 ***g*** for 5 min at 4 °C. Subsequently, the cells were washed in 1 mL ice‐cold PBS and then centrifuged at 1500 g for 5 min at 4 °C. The cells were then incubated with an anti‐human CD133 antibody Alexa Fluor^®^ 488 conjugate (Cell Signaling Technology) at room temperature for 1 h. The cells were then washed twice with wash buffer to remove excess antibody and analysed on a FACSCalibur flow cytometry system (BD Biosciences, San Jose, CA, USA) using cyflogic software V1.2.1 (Perttu Terho, Mika Korkeamaki, CyFlo Ltd., Turku, Finland). A total of 10^4^ events were collected for each sample.

### 2.8 Confocal microscopy

The cells were fixed in 4% paraformaldehyde in PBS and incubated with 0.1% Triton X‐100 for permeabilization. Immunolabelling with an anti‐βIII tubulin polyclonal antibody (Covance, Princeton, NJ, USA) or an anti‐GFAP (glial fibrillary acidic protein) monoclonal antibody (Thermo Scientific, Waltham, MA, USA) was performed by incubation at room temperature for 1 h. Secondary labelling was performed with Alexa Fluor 488‐conjugated secondary antibodies (Invitrogen). Coverslips were then mounted with DAPI‐containing Mowiol mounting medium (Sigma‐Aldrich). Imaging was performed with a Leica TCS SP5 laser scanning confocal microscope with las‐af imaging software using a 40X oil objective. The quantification of images was performed with imagej v1.8.0 software (NIH Image, Bethesda, MD, USA).

### 2.9 RNA extraction and reverse transcription quantitative polymerase chain reaction

Total cellular RNA was extracted from sensitive and resistant cells using the RNeasy Mini Kit (Qiagen, Hilden, Germany) according to the manufacturer's protocol. Total RNA (2–4 μg) underwent cDNA synthesis using SuperScript™ RT (Roche, Basel, Switzerland) according to the manufacturer's protocol. qPCR was performed in a 10 μL volume using SYBR‐Green PCR Master Mix (Takara Bio, Inc., Kusatsu, Japan) on a 7500 Real‐Time PCR System (Applied Biosystems Inc., Foster City, CA, USA) according to the manufacturer's protocols. PCR amplification was carried out using the following primer sequences: Nanog‐F 5′‐TTTGTGGGCCTGAAGAAAACT‐3′, Nanog‐R 5′‐AGGGCTGTCCTGAATAAGCAG‐3′; Oct4‐F 5′‐GACAGGGGGAGGGGAGGAGCTAGG‐3′, Oct4‐R 5′‐CTTCCCTCCAACCAGTTGCCCCAAAC‐3′; and ABCB1A‐F 5′‐TTGCTGCTTACATTCAGGTTTCA‐3′, ABCB1A‐R 5′‐AGCCTATCTCCTGTCGCATTA‐3′.

### 2.10 siRNA transfections

Cells were transfected in 1 mL OPTIMEM containing 4 μg Lipofectamine iMax (Invitrogen) with 100 nm AMPK‐specific small interfering RNA (siRNA) duplexes (Ambion‐Life Technologies, Carlsbad, CA, USA) or scrambled RNA (control) according to the manufacturer's protocols (Invitrogen). At 48 h after transfection, the medium was removed and replaced with DMEM. At the indicated time points after transfection, cells were used for MTT cell viability assays or western blot analysis.

### 2.11 Transient transfections

Plasmids encoding the full‐length human AMPK‐α1, AMPK‐β1 and AMPK‐γ1 were kindly provided by G. Hardie (University of Dundee, UK). Sensitive and resistant HepG2 and Huh7 cells were cotransfected with 5 μg recombinant α1 (pcDNA5‐FRT α1‐Flag), β1 (pCMV β1‐untagged) and γ1 WT (pcDNA5‐Flpln‐T10 γ1 WT‐Flag) plasmids using 5 μL Lipofectamine 3000 (Thermo Fisher) in antibiotic‐free medium and seeded into 12‐well plates. After 48 h of transfection, the transfection medium was replaced with another medium without serum, and the cells were maintained for 24 h and then assayed for cell viability. Protein expression was assayed by western blotting using anti‐FLAG antibodies (anti‐Flag M2 antibody, Sigma‐Aldrich). For tetracycline‐inducible plasmids, Flag‐tagged pcDNA5‐Flpln‐T10 protein expression was induced by the addition of 1 μg·mL^−1^ tetracycline in the transfection medium.

### 2.12 Animal studies

All animal experiments followed the ARRIVE guidelines and were carried out in accordance with the U.K. Animals (Scientific Procedures) Act, 1986, and associated guidelines, EU Directive 2010/63/EU for animal experiments. The procedure was approved by the Alcalá University Ethical Commission and by the Ethical Commission of the Comunidad de Madrid (procedure PROEX 241/15). All animal studies were conducted in accordance with the Spanish institutional regulation (RD 53/2013) for the housing, care and use of experimental animals and met the European Community directives regulating animal research. Recommendations made by the United Kingdom Coordinating Committee on Cancer Research (UKCCCR) were followed carefully. To assess the welfare of animals, a panel of 10 indicators were recorded each day. When adverse effects, pain or distress was observed in the animals (score of 15 of 40), a humane endpoint was applied.

Athymic nude‐Foxn1 (nu/nu) four‐week‐old mice were purchased from Envigo RMS (Barcelona, Spain) and housed in a laminar air flow cabinet under pathogen‐free conditions on a 12‐h light/dark schedule at 21–23 °C and 40‐60% humidity with access to food pellets and tap water ad libitum. Four animals were housed per cage. G Power analysis was used to calculate the sample size (Charan and Kantharia, 2013) according to our previous data and experience and considering a two‐tail effect and a significance level of 5%. Hepatocarcinoma tumours were induced in athymic mice by subcutaneous injection of 5 × 10^6^ HepG2 or HepG2SF1 cells. When tumours reached a volume of 70 mm^3^, mice were then randomly divided into experimental groups of 6 animals each, which received i.p. injections of 30 mg·kg^−1^ sorafenib (SF), 5 mg·kg^−1^ capsaicin or vehicle (DMSO) daily. Tumour size was measured daily and calculated using the formula V(mm^3^) = 1/2(length × width^2^). At the end of the study, the mice were sacrificed by placing them in a CO_2_ gas‐filled chamber, and the excised tumours were recovered and weighed. For the tumourigenic assay, hepatocarcinoma tumours were induced by subcutaneous injection of 2.5 × 10^6^, 5 × 10^6^, 10 × 10^6^ or 15 × 10^6^ HepG2 or HepG2SF1 cells on both sides of the mice. Tumour development was examined daily, and tumour volumes were calculated as indicated above.

### 2.13 Statistical analysis

Statistical significance was estimated with graphpad 6.0 (La Jolla, CA, USA) software using 1‐way or 2‐way ANOVA and Tukey's multiple comparison test or the unpaired Student's t‐test when indicated. Data are presented as the mean ± SD.

## 3 Results

### 3.1 Sorafenib resistance in HCC cells induces a stem‐like phenotype

Drug resistance has been frequently associated with the emergence of cancer stem cells (Phi *et al*., 2018). To study the resistance mechanisms in HCC cells, we induced sorafenib‐resistant cells by long‐term incubation of HepG2 and Huh7 HCC cell lines with step‐wise increasing concentrations of sorafenib (0.75–8 μm). After 12 months of exposure, the surviving cells that had routinely grown in the presence of 8 μM sorafenib were selected and designated as HepG2SF1 and Huh7SF1 cells.

**Figure 1:**
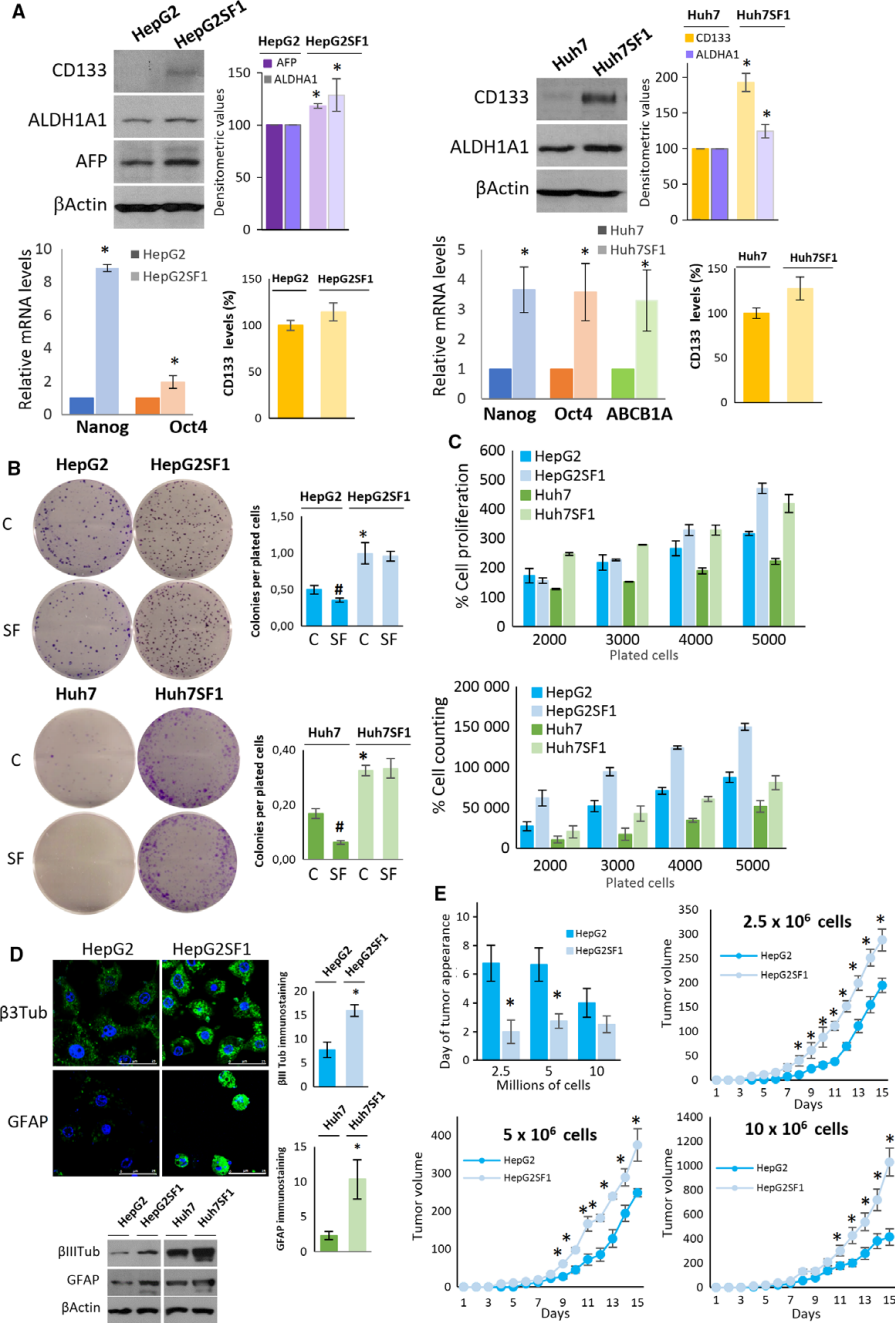
Long‐term treatment of HCC cells with sorafenib induces a stem‐like phenotype. (A) HepG2 and Huh7 cells were incubated with increasing concentrations of sorafenib for 12 months. The levels of stem cell markers were determined by western blot, qPCR and flow cytometry in parental HepG2 and Huh7 cells as well as in HepG2SF1 and Huh7SF1 cells treated long term with sorafenib. The qPCR data show the relative mRNA expression of actin, which was used as a housekeeping gene. Data represent the mean ± SD of seven independent experiments. (B) Colony‐forming capability (represented as the number of colonies formed/plated cells) of cells in the presence of vehicle (C) or 8 μm sorafenib (SF). (C) Cell proliferation determined by the MTT assay and cell counting of parental HepG2 and Huh7 and HepG2SF1 and Huh7SF1 cells treated long term with sorafenib. (D) Differentiation capability of HepG2SF1 and Huh7SF1 cells. Cells were grown in neuron differentiation media for 21 days, after which the expression of βIII tubulin was determined by immunofluorescence or western blot. Alternatively, cells were grown in glial differentiation media, and the expression of glial acidic fibrillary protein (GFAP) was determined by immunofluorescence or western blot. Scale bar of confocal images indicates 25 μm. Data are the mean ± SD of three independent experiments. (E) Tumourigenic potential of HepG2SF1 and Huh7SF1 cells. Nude mice were inoculated with 2.5, 5 or 10 million HepG2 or HepG2SF1 cells, and tumour size was examined daily. Figure represents the frequency of tumour development (upper left panel) and the growth curves of the tumours over 15 days (mean ± SEM,* n *=* *6). *P ≤ 0.01 compared by Student's *t*‐test.

To investigate whether long‐term sorafenib treatment induced CSC differentiation in HCC cultures, we analysed the expression of the cell membrane protein CD133, which was recently identified as a stem cell marker in HCC (Castelli *et al*., 2017). According to previous data (Suetsugu *et al*., 2006), parental HepG2 cells do not express CD133 (Fig. 1A). Likewise, in Huh7 cells, CD133 is barely expressed (Fig. 1A). Interestingly, in HepG2SF1 cells treated with sorafenib long term, there was a slight increase in CD133 expression, whereas in Huh7SF1 cells, a notable increase in CD133 expression was observed (Fig. 1A). Flow cytometry determination of CD133 confirmed its increase in HepG2SF1 and Huh7SF1 cells (Figs 1A and S1). As CD133 was poorly detected in HepG2 cells, we examined the embryonic protein alpha fetoprotein (ΑFP), which has also been proposed as a stem cell marker and is clearly detected in HepG2 cells (but not in Huh7 cells). As shown in Fig. 1A, ΑFP expression increased in HepG2SF1 cells treated with sorafenib long term, indicating the acquisition of a stem‐like phenotype. To further investigate this hypothesis, we examined the expression of other stem cell‐associated genes, such as aldehyde dehydrogenase isoform 1A1 (ALDH1A1), a cytosolic enzyme that metabolizes reactive aldehydes and reactive oxygen species. Due to its role in metabolism, this detoxifying enzyme can confer cellular protection against cytotoxic drugs such as sorafenib. ALDH1A1 expression was increased in HepG2SF1 and Huh7SF1 cells (Fig. 1A). To examine the time course of the appearance of these stem markers, we analysed their expression 6 months after sorafenib treatment, and we observed that although A and CD133 in HepG2SF1 cells and CD133 in Huh7SF1 cells could be appreciated at 6 months, their expression was much lower than that at 12 months (Fig. S1). The enzyme ALDH1A1 was increased only after 12 months of sorafenib treatment (Fig. S1). Therefore, we continued the study with cells treated for 12 months with sorafenib.

We next analysed the expression of ABCB1A or p‐glycoprotein, a member of the multidrug resistant transporters that actively extrudes a variety of hydrophobic amphipathic drugs from the cell. This transporter was only analysed in Huh7 cells because we were not able to detect ABCB1A in HepG2 cells, which is probably due to the low expression of this gene in this cell line. Finally, we examined the transcription factors Nanog and octamer‐binding transcription factor 4 (Oct4), which are usually expressed in embryonic stem cells, where they are involved in pluripotency regulation, proliferation and renewal. These transcription factors have also been used to detect cancer stem cell subpopulations (Iv Santaliz‐Ruiz *et al*., 2014; van Schaijik *et al*., 2018; Zheng *et al*., 2013). The increased expression of all the genes analysed in sorafenib‐resistant cells (Fig. 1A) indicated that long‐term treatment of HCC cells with sorafenib induced the expression of proteins involved in drug resistance and pluripotency, characteristic of cancer stem cells.

Then, we investigated whether long‐term sorafenib treatment led to other features of cancer stem cells. Cancer stem cells are capable of forming large colonies through clonal expansion from a single cell, and clonogenic activity has been considered an important indicator of undifferentiation (Rajendran and Jain, 2018). In agreement with this notion, HepG2SF1 and Huh7SF1 cells had a higher capacity of forming colonies than their parental cells (Fig. 1B). Notably, HepG2SF1 and Huh7SF1 cells formed colonies even when treated with sorafenib, whereas their parental cells exhibited decreased numbers of colonies in the presence of this drug (Fig. 1B). In addition, HepG2SF1 and Huh7SF1 cells showed a higher proliferation rate than HepG2 and Huh7 cells (Fig. 1C). One of the critical properties of CSCs is their potential to differentiate into unlimited heterogeneous populations of cancer cells (Desai *et al*., 2018). To investigate the differentiation potential of the long‐term sorafenib‐treated cells, HepG2, Huh7, HepG2SF1 and Huh7SF1 cells were incubated in neuronal differentiation or glial differentiation media for 15 days. Afterwards, differentiation into neurons was assessed by determining the expression of the specific neuronal marker βIII tubulin and differentiation into astrocytes by the expression of glial fibrillary acidic protein (GFAP). As shown in Fig. 1D, the incubation of cells with neuronal differentiation media induced a notorious increase in the expression of βIII tubulin in HepG2SF1 and Huh7SF1 cells compared with their parental cells (Fig. 1D). Likewise, the expression of GFAP increased in HepG2SF1 and Huh7SF1 cells when incubated in glial differentiation media (Fig. 1D). We then examined the tumourigenic potential of the stem‐like cells. According to the European normative on the reduction of animals in research (3 Rs principle), *in vivo* experiments were carried out only in the HepG2 cell line. To this end, HepG2 or HepG2SF1 cells were transplanted to generate xenograft‐derived tumours in mice. The frequencies of tumour formation were measured after the inoculation of nude mice with either 2.5, 5 or 10 million cells. As shown in Fig. 1E, tumours derived from HepG2SF1 cells appeared earlier than those derived from HepG2 cells, confirming that the stem‐like cells had an enhanced capacity to form xenograft‐derived tumours compared to their parental cells. Likewise, tumours derived from stem‐like cells grew faster than tumours derived from parental cells (Fig. 1E). Taken together, these results indicate that HepG2SF1 and Huh7SF1 cells exhibit phenotypic characteristics of cancer stem cells.

**Figure 2:**
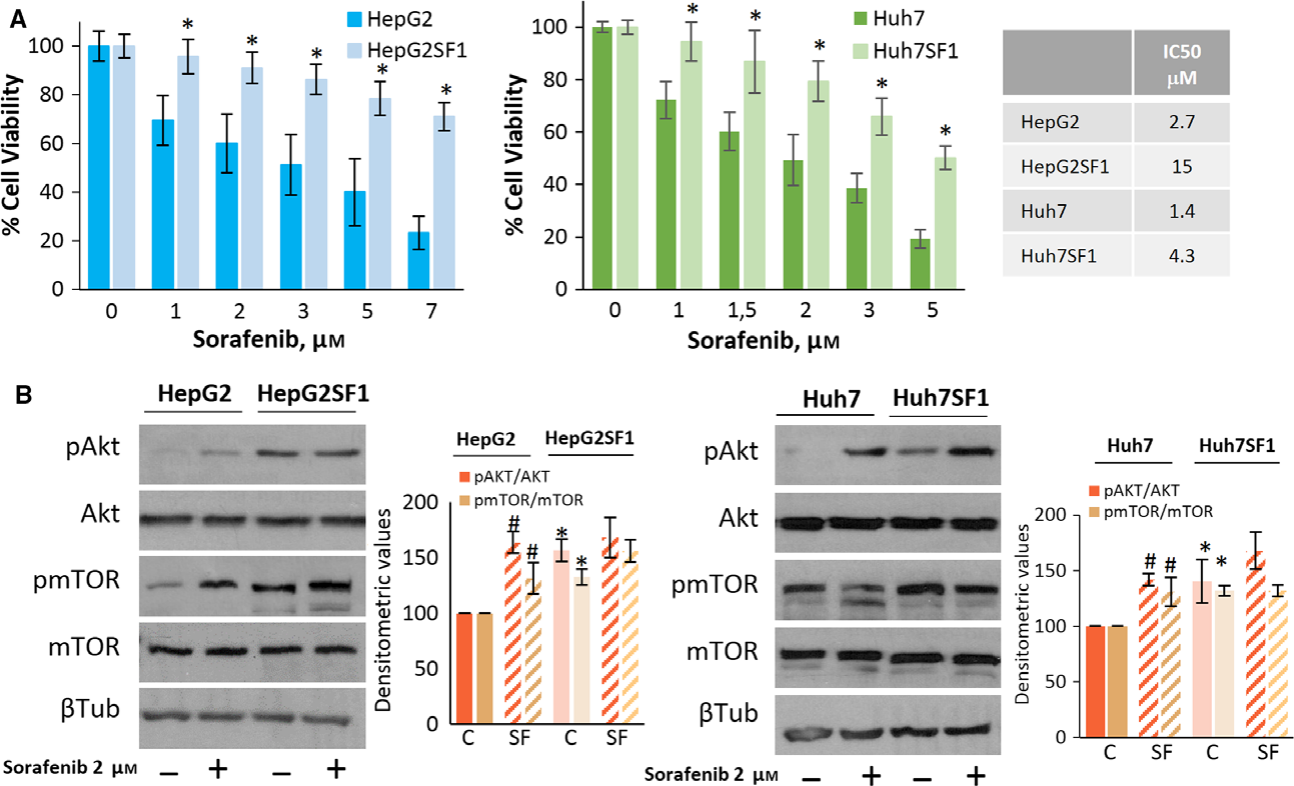
Chemoresistance of HepG2SF1 and Huh7SF1 cells. (A) Cells were treated with sorafenib at the indicated concentrations for 24 h. Cell viability was determined by the MTT assay and is expressed as the percentage of the control (DMSO treatment). The table shows the IC50 values of sorafenib in the four cell lines. Experiments were run in triplicate and carried out three times on separate occasions. (B) The levels of phosphorylated Akt and mTOR proteins and their total forms were determined by western blot. β‐Tubulin (βTub) is shown as a loading control. A representative image of four different experiments is shown. Densitometric values (mean ± SD,* n *=* *4) relative to controls are shown on the right. *P <* *0.005 significant difference between stem‐like and parental cells and ^#^ *P *<* *0.005 between treated and nontreated cells by two‐way ANOVA and Tukey's multiple comparisons test.

To investigate whether these stem‐like cells were resistant to sorafenib, cells were treated in the absence of serum with 0, 1, 2, 3, 5 and 7 μm sorafenib for 24 h. The results in Fig. 2A show that HepG2SF1 and Huh7SF1 cells could tolerate higher concentrations of sorafenib in the absence of serum compared to parental cells, with an IC50 fivefold and threefold higher than that of HepG2 and Huh7 cells, respectively (Fig. 2B).

Dysregulated signalling of the PI3K/Akt/mTOR pathway has been associated with resistance to several different chemotherapeutic agents, including sorafenib (Zhu *et al*., 2017). To address the status of this pathway, we examined the total and phosphorylation levels of Akt and mTOR in sensitive and sorafenib‐resistant HCC cells. As shown in Fig. 2B, HepG2SF1 and Huh7SF1 stem‐like cells had higher Akt and mTOR phosphorylation levels compared to sensitive cells, which is in good agreement with previous reports demonstrating the activation of this signalling pathway in chemotherapy‐resistant cells (Dong *et al*., 2017; Lindblad *et al*., 2016).

### 3.2 AMPK inhibition induces the acquisition of a stem‐like phenotype

**Figure 3:**
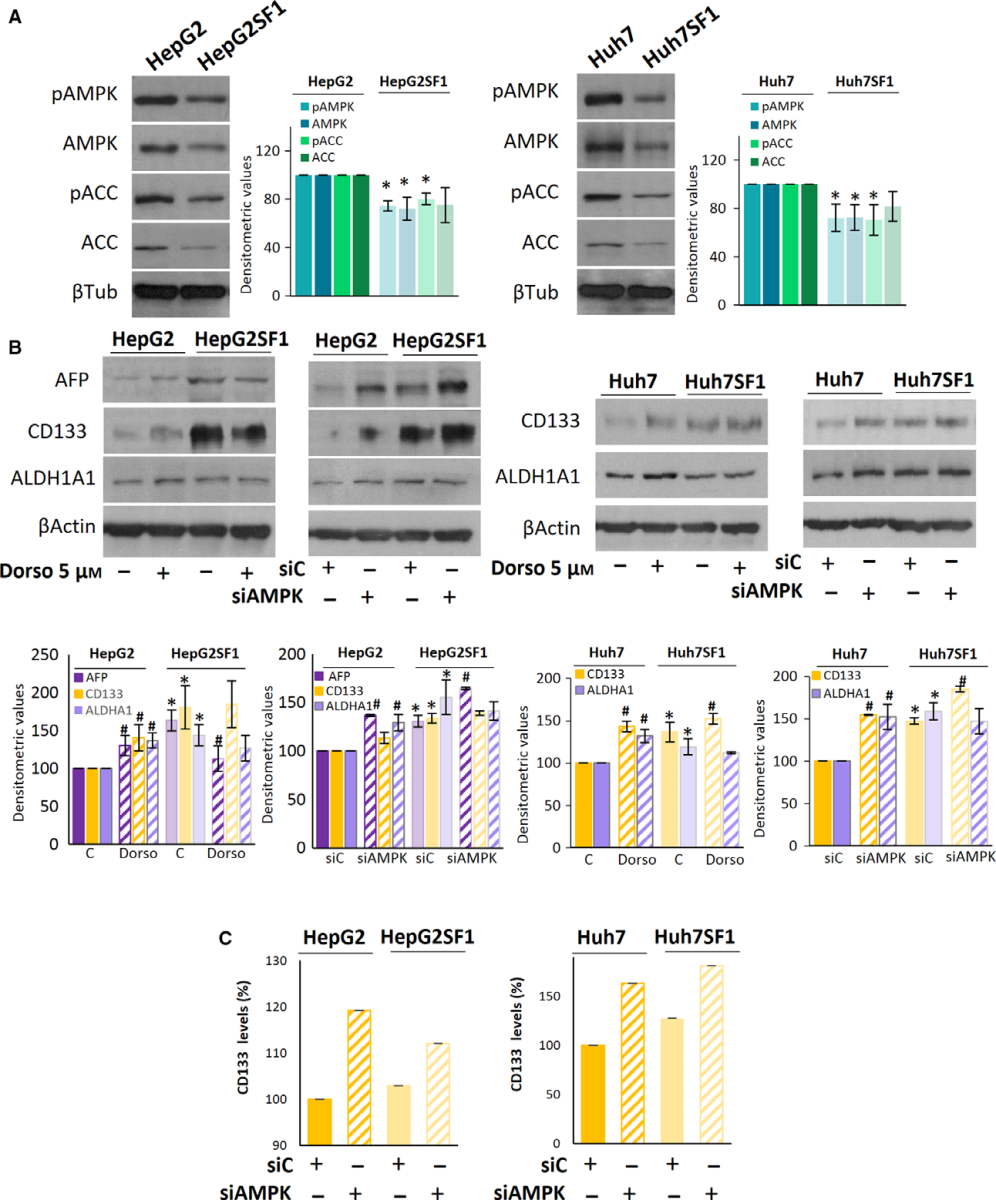
The AMPK inhibition induces stemness in HCC cells. (A) The levels of phosphorylated and total forms of AMPK and ACC in parental (HepG2 and Huh7) and stem‐like HepG2SF1 and Huh7SF1 cells were determined by western blot**.** β‐Tubulin (βTub) is shown as a loading control. A representative image of four different experiments is shown. Densitometric values (mean ± SD,* n *=* *4) relative to controls are shown on the right. (B) Effect of the AMPK inhibitor dorsomorphin (Dorso) or AMPK knockdown with an siRNA on stem cell marker expression in parental (HepG2 and Huh7) and stem‐like (HepG2SF1 and Huh7SF1) cells as determined by western blot. β‐Actin is shown as a loading control. A representative image of three different experiments is shown. Densitometric values (mean ± SD,* n *=* *3) relative to controls are shown below. (C) The levels of CD133 were determined by flow cytometry in AMPK‐knockdown cells.

We recently showed that AMPK activation increases HCC cell sensitivity to sorafenib (Bort *et al*., 2017). Consequently, we wondered whether AMPK inhibition was involved in sorafenib‐acquired resistance and in the stem‐like cell phenotype of HepG2SF1 and Huh7SF1 cells. To this end, we first determined the expression levels of AMPK and its phosphorylation at the activation site Thr172, as well as the phosphorylation and levels of its downstream target, acetyl‐CoA carboxylase (ACC), in HepG2SF1 and Huh7SF1 cells. Decreased phosphorylation and expression of both AMPK and ACC were clearly observed in sorafenib‐resistant cells compared with their parental cells (Fig. 3A). Therefore, we examined the effect of AMPK downregulation on the expression of stem cell markers in HepG2SF1 and Huh7SF1 cells. To this end, cells were either treated with the AMPK inhibitor dorsomorphin or transfected with an siRNA targeting AMPK. As shown in Fig. 3B, AMPK inhibition or knockdown increased the expression of ΑFP, CD133 and ALDH1A1 in sensitive HepG2 cells and of CD133 and ALDH1A1 in Huh7 cells. The increase in CD133 in AMPK‐depleted cells was further confirmed by flow cytometry (Figs 3C and S2). These results suggest a negative role for AMPK in the stemness of HCC cells.

**Figure 4:**
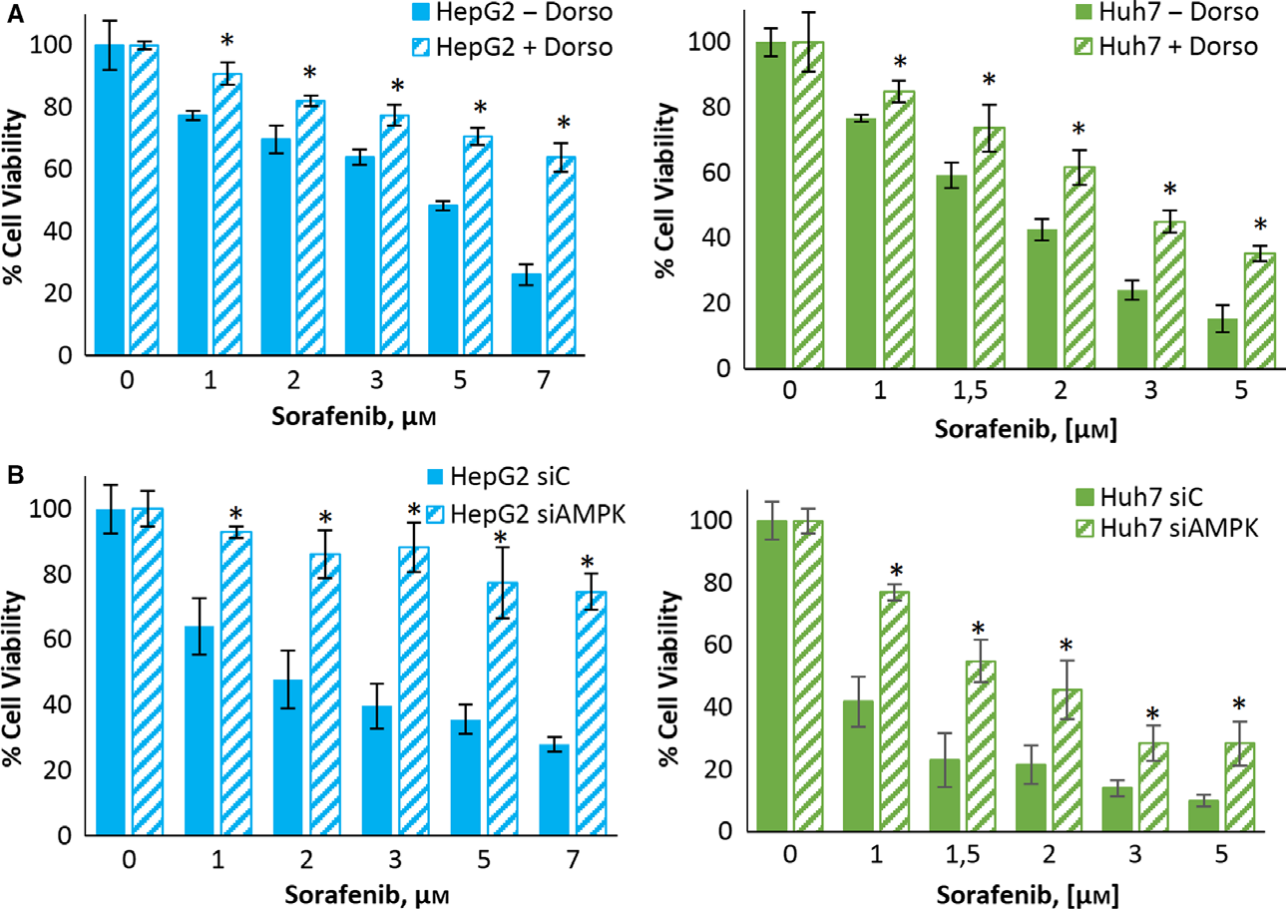
The AMPK inhibition induces sorafenib resistance in HCC cells. (A) Effect of dorsomorphin on the viability of HepG2 and Huh7 cells treated with sorafenib. Cells were treated with or without 5 μm dorsomorphin and sorafenib at the indicated concentrations for 24 h. Cell viability was determined by the MTT assay and is expressed as the percentage of the control (DMSO treatment). (B) Effect of AMPK knockdown on the viability of HepG2 and Huh7 cells treated with sorafenib. Cells were transfected with either siRNA‐control or an AMPK‐selective siRNA for 24 h and then treated with sorafenib at the indicated concentrations for 24 h. Cell viability was determined by the MTT assay and is expressed as the percentage of the control (DMSO treatment). Experiments were run in triplicate and carried out three times on separate occasions (data are the mean ± SD). *P <* *0.005 significant difference between resistant and control cells and ^#^ *P *<* *0.005 between treated and nontreated cells by two‐way ANOVA and Tukey's multiple comparisons test.

To address the relevance of AMPK downregulation in the resistance to sorafenib, we treated sensitive cells with the AMPK inhibitor dorsomorphin and examined cell viability. The inhibition of AMPK reduced sorafenib‐induced cell death in sensitive HepG2 and Huh7 cells (Fig. 4A) but had no effect on stem‐like sorafenib‐resistant cells (Fig. S3). Likewise, AMPK knockdown with an siRNA completely abolished the antiproliferative effect of sorafenib in sensitive parental cells, with no effect on stem‐like cells (Figs 4B and S3). These findings suggest that AMPK activity is necessary for sorafenib‐induced cell death and that AMPK downregulation makes cells refractory to sorafenib.

### 3.3 AMPK upregulation decreases the expression of stem cell markers in HepG2SF1 and Huh7SF1 cells

**Figure 5:**
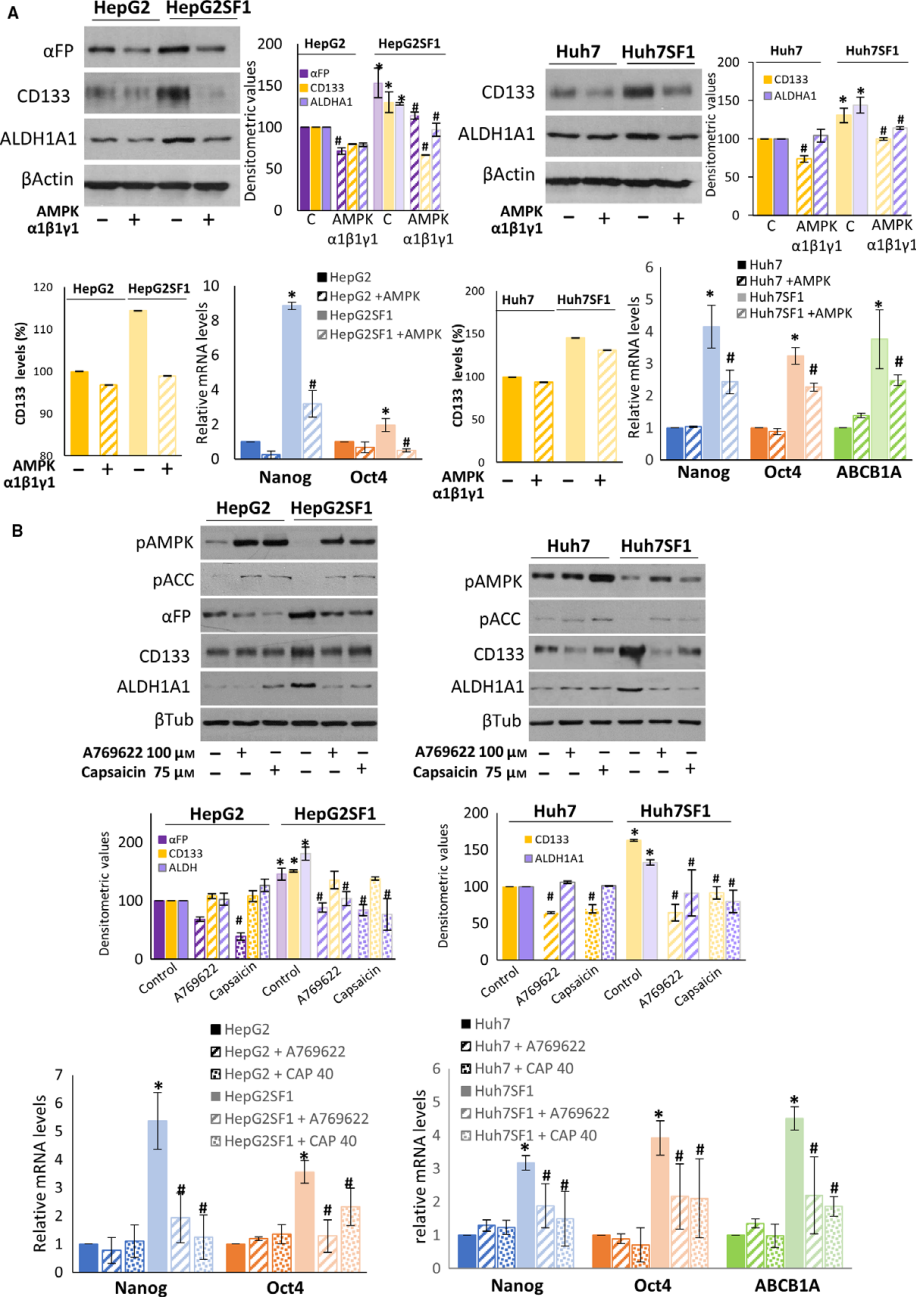
The AMPK upregulation decreases the expression of stem cell markers in HepG2SF1 and Huh7SF1 cells. (A) The levels of stem cell markers in parental HepG2 and Huh7 cells and stem‐like HepG2SF1 and Huh7SF1 cells transfected with AMPK α1β1γ1 were determined by western blot and qPCR. β‐Actin is shown as a loading control in western blots. A representative image of three different experiments is shown. Densitometric values (mean ± SD,* n *=* *3) relative to controls are shown on the right. The qPCR data show the relative mRNA expression of actin, which was used as a housekeeping gene. (B) The levels of pAMPK, pACC and stem cell markers were determined by western blot and qPCR in cells treated with the AMPK activator A‐769662 and capsaicin. A representative image of three different experiments is shown. Densitometric values (mean ± SD,* n *=* *3) relative to controls are shown below. The qPCR data show the relative mRNA expression of actin, which was used as a housekeeping gene. *P <* *0.005 significant difference between resistant and control cells by two‐way ANOVA and Tukey's multiple comparisons test, ^#^ *P *<* *0.005 significant difference between AMPK‐transfected and nontransfected cells (panel A) or between control and treated cells (panel B).

To further explore the functions of AMPK as a drug resistance suppressor, we investigated whether AMPK upregulation could modify the expression of drug resistance and stem cell markers in sorafenib‐refractory cells. For this purpose, we first induced AMPK expression by transient transfection with plasmids containing the AMPK subunits α1, β1 and γ1 (Fig. S4). As shown in Fig. 5A, AMPK transfection notably decreased the expression of CD133, ALDH1A1 and alpha fetoprotein in sorafenib‐resistant cells, as determined by western blot. The determination of CD133 by flow cytometry confirmed the decrease in this stem cell marker in HepG2SF1 cells transfected with AMPK (Figs 5A and S2). Likewise, the levels of the drug efflux transporter ABCB1A and the embryonic genes OCT4 and NANOG decreased in AMPK‐transfected cells (Fig. 5A).

We then treated resistant cells with the well‐known AMPK activator A‐769662 and capsaicin, which, as we previously showed, activates AMPK in HepG2 cells (Bort *et al*., 2017). As shown in Fig. 5B, A‐769662 and capsaicin activated AMPK in both sensitive and resistant cells. Treatment of HCC cells with A‐769662 markedly decreased the expression of ΑFP, CD133 and ALDH1A1 in HepG2SF1 cells as well as the levels of CD133 and ALDH1A1 in Huh7SF1 cells. The effect of capsaicin on CD133 levels was similar, although not as strong. Similarly, A‐769662 and capsaicin decreased the expression of Nanog, OCT4 and ABCB1A in the stem‐like cells (Fig. 5B).

### 3.4 AMPK overexpression or activation restores the sensitivity to sorafenib in stem‐like cells

**Figure 6:**
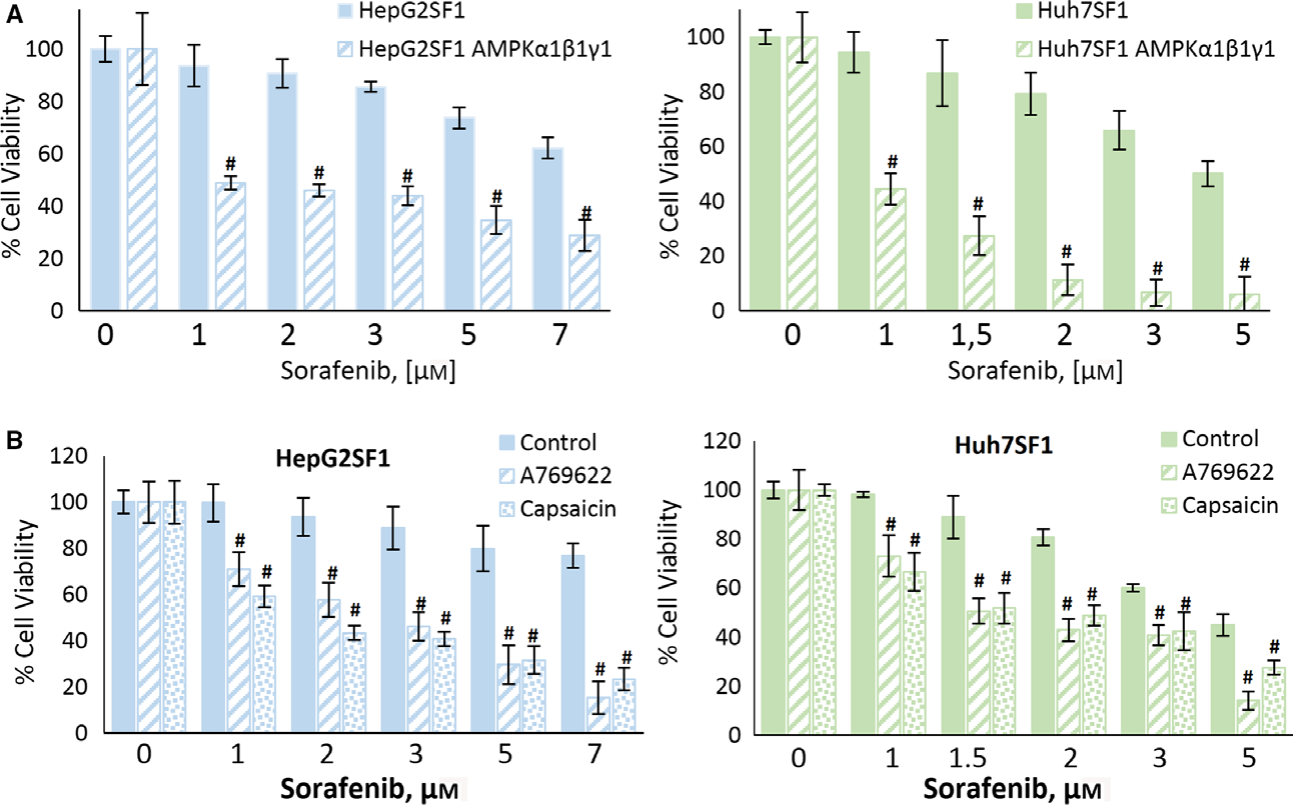
The AMPK upregulation restores sorafenib sensitivity in HCC cells. (A) Effect of the transient expression of AMPKα1β1γ1 on cell viability in HepG2SF1 and Huh7SF1 cells treated with increasing concentrations of sorafenib. Cells were treated with sorafenib at the indicated concentrations for 24 h. Cell viability was determined by the MTT assay and is expressed as the percentage of the control (DMSO treatment). Experiments were run in triplicate and carried out three times on separate occasions. (B) Effect of the AMPK activator A‐769662 and capsaicin on cell viability in HepG2SF1 and Huh7SF1 cells treated with increasing concentrations of sorafenib. Cells were treated as described above. Experiments were run in quadruplicate and carried out three times on separate occasions (data are the mean ± SD). #*P *<* *0.005 significant difference between AMPK‐transfected and nontransfected cells (panel A) or between control and treated cells (panel B).

We next examined whether AMPK overexpression could resensitize stem‐like cells to sorafenib. Interestingly, the overexpression of AMPK in HepG2SF1 and Huh7SF1 cells resulted in sorafenib‐induced cell death at levels comparable with those of sensitive cells (Fig. 6A). The overexpression of AMPK in sensitive cells did not modify the effect of sorafenib (Fig. S4). In agreement with these results, the treatment of stem‐like cells with compound A‐769662 or capsaicin resensitized cells to sorafenib (Fig. 6B), whereas it had little effect on sensitive cells (Fig. S4).

### 3.5 Potential mechanisms involved in the AMPK effect

**Figure 7:**
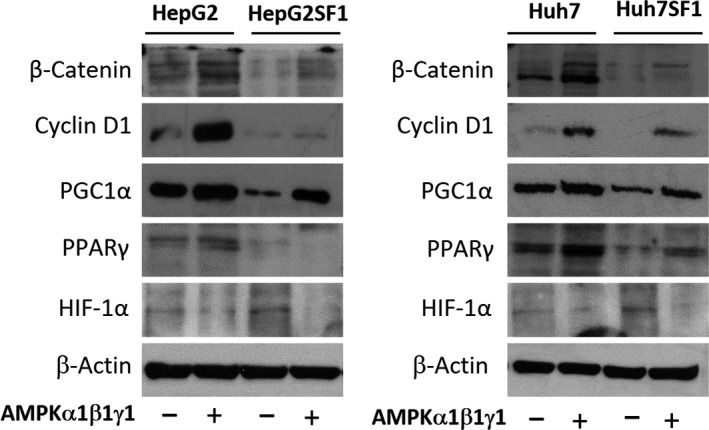
The AMPK transfection counteracts β‐catenin, cyclin D1, PGC1α and PPARγ downregulation and HIF‐1α upregulation in stem‐like cells. HepG2 and Huh7 cells and stem‐like HepG2SF1 and Huh7SF1 cells were transfected with AMPK α1β1γ1, and the levels of β‐catenin, cyclin D1, PGC‐1α, PPARγ and HIF‐1α were determined by western blot. β‐Actin is shown as a loading control. A representative image of two different experiments is shown.

To elucidate the potential mechanisms underlying the AMPK effect on stem‐like cells, we analysed some of the signalling pathways associated with development and differentiation in HCC cells transfected with AMPK. Previous studies have reported that the Wnt/β‐catenin signalling pathway is associated with the control of stem cell differentiation (Valkenburg *et al*., 2011). The activation of this pathway causes the accumulation of β‐catenin in the cytosol, which is translocated to the nucleus, where it binds to LEF/TCF transcription factors and regulates the expression of target genes, including genes involved in differentiation and the cell cycle. To investigate whether the Wnt/β‐catenin pathway was activated in HCC stem‐like cells, we determined, by western blot, the levels of β‐catenin and cyclin D1, a downstream target of β‐catenin. The results in Fig. 7 show that β‐catenin and cyclin D1 levels were decreased in HepG2SF1 and Huh7SF1 cells compared to their parental cells, while they were increased in AMPK‐transfected cells. It has been demonstrated that β‐catenin, through repression of the transcription factor TCF7L2, upregulates the expression of peroxisome proliferator‐activated receptor gamma coactivator‐1 alpha (PGC‐1α), which has been shown to be involved in hepatocyte differentiation (Jiao *et al*., 2017; Wanet *et al*., 2017). In contrast, AMPK can directly phosphorylate and activate PGC‐1α and regulate its expression (Colombo and Moncada, 2009). Therefore, we analysed PGC1α levels in HCC cells and observed that this coactivator was downregulated in stem‐like HepG2SF1 and Huh7SF1 cells (Fig. 7), in concordance with AMPK levels in those cells. In line with this, the levels of peroxisome proliferator‐activated receptor gamma (PPARγ) were decreased in stem‐like cells (Fig. 7). Interestingly, the overexpression of AMPK by transient transfection increased the levels of PGC1α and PPARγ (Fig. 7).

Recent findings have indicated that the hypoxia‐inducible factors HIF‐1α and HIF‐2α play critical roles in the gain of more malignant phenotypes by cancer stem cells (Mimeault and Batra, 2013). More specifically, it has been observed that hypoxia and enhanced HIF‐1α and HIF‐2α expression and activity, which frequently occur during tumour progression, may result in the upregulation of different stemness gene products that increase tumourigenicity (Heddleston *et al*., 2009; Ma *et al*., 2011; Mimeault and Batra, 2013). Importantly, it has been described that the downregulation of AMPK under normoxia induces a sustained increase in HIF‐1α and cooperates with c‐myc to increase tumourigenesis (Faubert *et al*., 2013). Thus, we determined the levels of HIF‐1α in HepG2SF1 and Huh7SF1 cells transfected with AMPK. As shown in Fig. 7, the stem‐like cells HepG2SF1 and Huh7SF1 had increased levels of HIF‐1α, suggesting that the depletion of AMPK could reverse the inhibition of this transcription factor, which might be involved in the upregulation of stem cell markers observed in these cells (Fig. S5). Notably, the levels of HIF‐1α decreased in AMPK‐transfected cells, in agreement with the observed decrease in stem cell markers under these conditions. All these results indicate that AMPK, by downregulating PGC‐1α and PPARγ and by upregulating HIF‐1α, could induce HCC stem cell reprogramming and restore sorafenib sensitivity.

### 3.6 AMPK activation reduces the tumour growth of stem‐like cells *in vivo*

**Figure 8:**
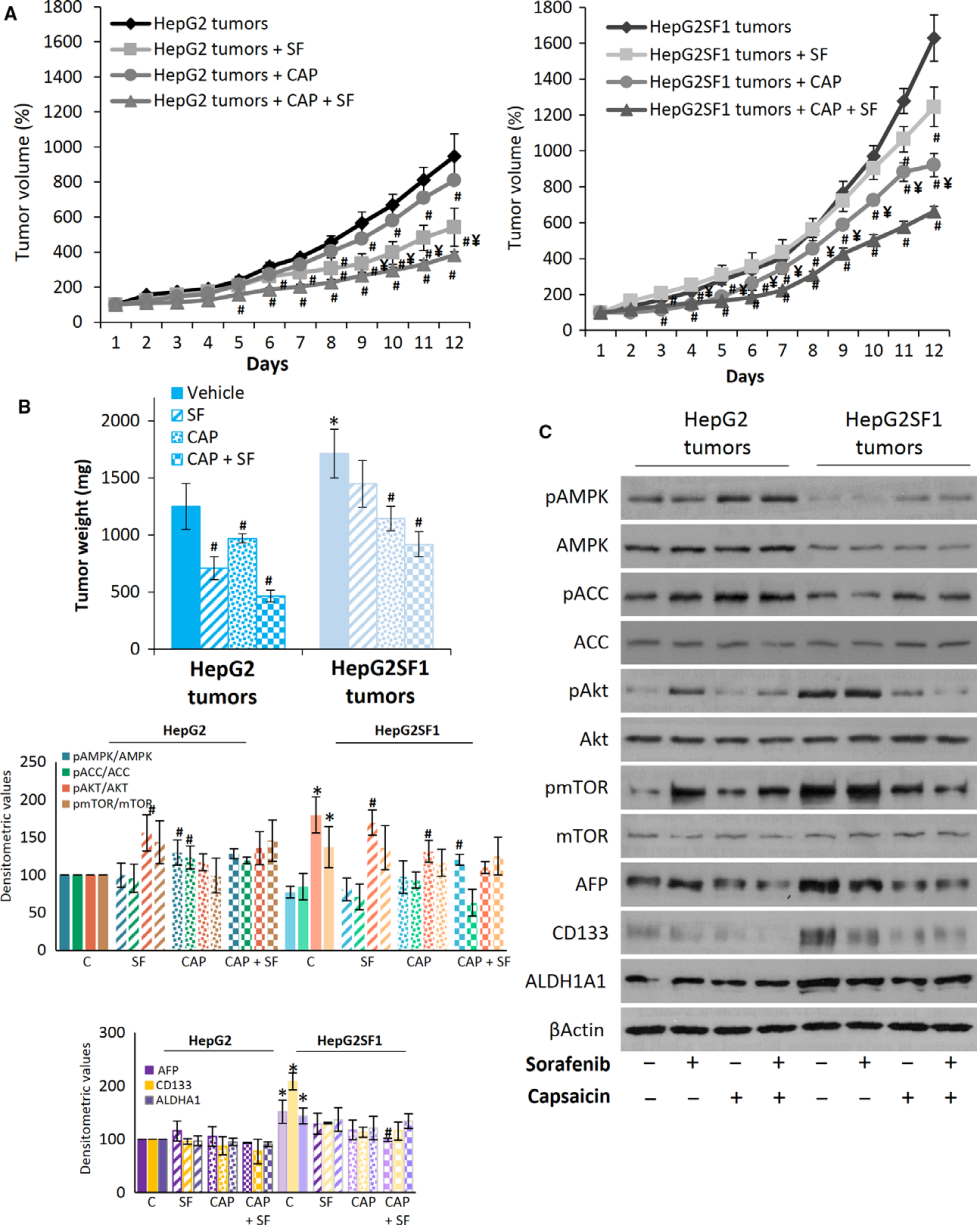
*In vivo* activity of sorafenib and capsaicin in stem‐like cells. (A) The growth curves of HepG2 and HepG2SF1 cells as tumour xenografts in nude mice treated with vehicle (diamonds), 30 mg·kg^−1^·day^−1^ sorafenib (squares), 5 mg·kg^−1^·day^−1^ capsaicin (circles) or both (triangles). Data are shown as the mean ± SEM;* n *=* *6. (B) Tumour weights at the end of treatment (mean ± SEM). (C) The levels of proteins in the tumours dissected at the end of the treatment were determined by western blot. β‐Actin is shown as a loading control. A representative image of one of the six tumours of each treatment is shown. Densitometric values (mean ± SD,* n *=* *6) relative to controls are shown on the left. *P <* *0.005 significant difference between resistant tumours and sensitive tumours; ^#^ *P *<* *0.005 between treated and nontreated tumours and ^¥^ *P *<* *0.005 between capsaicin‐ and sorafenib‐treated tumours by two‐way ANOVA and Tukey's multiple comparisons test.

To further assess the effects of sorafenib resistance on tumour growth *in vivo*, HepG2 or HepG2SF1 cells were inoculated in mice. Once the tumour size reached 70 mm^3^, the animals were randomly divided into groups and treated with vehicle, 30 mg·kg^−1^ sorafenib, 5 mg·kg^−1^ capsaicin or both compounds simultaneously for 12 days. We treated the mice with capsaicin since compound A769662 has shown poor bioavailability *in vivo* (Cool *et al*., 2006), and we have previous data on the *in vivo* AMPK activation by capsaicin (Bort *et al*., 2017). Tumour volumes were calculated daily. After sacrifice, the size and weight of the tumour were measured. The stem‐like HepG2SF1 cells had greater tumourigenicity than their parental HepG2 cells, since the tumours reached 70 mm^3^ twice as fast (data not shown). As shown in Fig. 8A, HepG2SF1 tumours grew clearly faster, especially in the last 5 days, when tumours grew exponentially. Moreover, sorafenib‐treated HepG2SF1 tumours grew at a similar rate as vehicle‐treated HepG2SF1 cells, further confirming the drug resistance developed by these cells. However, as expected, sorafenib reduced tumour growth in HepG2‐sensitive tumours (Fig. 8A). Notably, capsaicin was very efficient in reducing the tumour growth of stem‐like cells, which grew at a similar rate as that of parental cells (Fig. 8A). Likewise, stem‐like tumour weight at the end of the treatment was not significantly different between vehicle‐ and sorafenib‐treated mice, whereas it was significantly reduced by capsaicin (Fig. 8B). By contrast, the weights of sensitive tumours treated with sorafenib were significantly reduced compared with the vehicle‐treated animals.

Molecular analyses of sorafenib‐treated xenografts corroborated our findings in cell cultures. Immunoblot analysis of tumour lysates showed that tumours developed from stem‐like HepG2SF1 cells had increased expression of AFP, CD133 and ALDH1A1 as well as upregulation of the Akt/mTOR axis and lower expression of pAMPK, AMPK, pACC and ACC (Fig. 8C). Sorafenib treatment of HepG2 tumours slightly increased the expression of ΑFP, while sorafenib treatment of stem‐like tumours reduced the expression of CD133 but did not induce a significant modification of AFP or ALDH1A1. Interestingly, treatment of mice with capsaicin and especially with the combination of capsaicin and sorafenib activated AMPK and markedly reduced the expression of AFP, CD133 and ALDH1A1 (Fig. 8C), suggesting AMPK activation as a strategy to reduce stem‐like properties and resistance *in vivo*.

## 4 Discussion

Sorafenib resistance remains a major problem for the effective treatment of hepatocellular carcinoma because sorafenib is the only standard clinical treatment against the advanced form of this disease (Chen *et al*., 2015a). The therapeutic benefit of this compound is limited, and invariably, tumour progression reappears (Le Grazie *et al*., 2017). Therefore, it is necessary to identify signalling pathways that promote drug resistance and to explore potential strategies to overcome resistance to find more effective therapies. In this study, we analysed the role of AMP‐activated kinase in the development of the cancer stem cell phenotype in two HCC cell lines. HepG2 and Huh7 cells grown in the presence of sorafenib for 12 months showed higher expression of drug resistance (ALDH1A1, ABCB1A) and stem‐related (CD133, OCT4, Nanog, alpha fetoprotein) genes and had higher clonogenic capacity, a higher proliferation rate and differentiation ability and increased tumourigenic potential, suggesting that sorafenib resistance induced or selected stem‐like cells. Although the biological function of CD133 is not well understood, it currently serves as a useful marker for liver cancer stem cells. Studies by Ma *et al*. (2008a) found that CD133‐positive HCC cells possessed greater colony‐forming efficiency, a higher proliferative output and a greater ability to form tumours *in vivo*. The tumourigenic potential of those HCC cells correlated with the expression of both CD133 and ALDH (Ma *et al*., 2008a). Moreover, the overexpression of CD133 in 127 HCC specimens was associated with a poor prognosis (Dai *et al*., 2018). Recent results have shown that CD133 may interact with the regulatory subunit of PI3K, stabilizing AKT signalling and conferring CSC‐like properties to hepatocellular carcinoma (Jang *et al*., 2017a,2017b). Our results are in good agreement with these observations, as CD133‐positive cells presented higher proliferative and tumourigenic potential. In addition, we show that long‐term treatment of HCC cells with increasing doses of sorafenib induces the expression of pluripotency genes and converts them into highly aggressive and resistant cells. In agreement with our results, previous data showed that Nanog^+^ Huh7 cells exhibited increased chemoresistance, self‐renewal, sphere formation ability and *in vivo* tumour incidence compared to Nanog^−^ Huh 7 cells (Shan *et al*., 2012) when considering the Oct4 and Nanog transcription factors as gatekeepers of pluripotency (Lee *et al*., 2017; Seymour *et al*., 2015). In our study, the elevated expression of stem cell genes in HepG2SF1 and Huh7SF1 cells indicated that sorafenib resistance in HCC cells was associated with an increase in the CSC subpopulation and suggests the enrichment in CSCs as the underlying cause of resistance to sorafenib treatment in HCC.

Interestingly, these cells were refractory to sorafenib‐induced cell death and showed overactivation of the Akt/mTOR pathway. Our finding that sorafenib resistance was associated with Akt/mTOR upregulation is in line with other previous studies (Jilkova *et al*., 2018; Zhai *et al*., 2014; Zhu *et al*., 2017) and reveals the importance of this pathway in drug resistance.

The AMPK signalling pathway is a master regulator of cellular energy homeostasis and metabolism whose role in cancer is beginning to be revealed. There is clinical evidence that the regular use of pharmacological activators of AMPK to treat type 2 diabetes provides protection against the development of cancer (Hardie, 2015). Metformin impairs oncogenic signalling pathways such as Akt/mTOR and receptor tyrosine kinase (Saini and Yang, 2018), providing a proof of concept about the inhibitory role of activated AMPK in cell proliferation. In this study, we explored whether AMPK was involved in the drug resistance of HCC. Our results showed that in sorafenib‐resistant HCC cells, there was a decrease in the phosphorylation, expression and activation of AMPK. A recent study showed that sorafenib activated AMPK indirectly by inhibiting mitochondrial function in HEK293 cells (Ross *et al*., 2017). The sorafenib doses used in the previous study were higher than those used in the current study (10‐30 μm), and the treatment times were shorter (1 h). Nevertheless, we observed a decrease in AMPK phosphorylation in our model when cells acquired resistance to sorafenib. Interestingly, AMPK upregulation, either by pharmacological activation or by transfection, was very successful not only in restoring sorafenib sensitivity but also in reducing the expression of most cancer stem markers analysed. Conversely, AMPK inhibition or AMPK knockdown induced sorafenib resistance in sensitive HCC cells. These results indicate that chemoresistance to sorafenib can be overcome by AMPK upregulation, suggesting a promising clinical therapeutic strategy to fight resistant HCC. This notion is in accordance with recent findings demonstrating that resistance to sorafenib may have a reversible phenotype (Kuczynski *et al*., 2015).

Based on our aforementioned findings, we further confirmed the relationship between AMPK and sorafenib resistance in a mouse xenograft model. *In vivo* studies demonstrated that HepG2SF1 cells possessed higher tumourigenic potential and that tumours generated from these cells displayed stronger resistance to sorafenib. In addition, HepG2SF1 tumours had lower expression of AMPK and higher expression of stem cell markers.

Although the role of AMPK in chemoresistance is unknown, Zeng *et al*. (2018) recently showed that the inhibition of LKB1 and consequently AMPK induced docetaxel resistance in prostate cancer cells. Moreover, additional findings support the concept that AMPK activation could hinder the development of CSCs. For instance, AMPK activation inhibits the sphere‐forming ability of the CSC subpopulation in breast, pancreatic and ovarian cancer models (Saini and Yang, 2018). In line with this notion, a recent study demonstrated that CSCs from diabetic patients complicated with colorectal carcinoma and treated with metformin showed lower proliferation and higher rates of apoptosis than those of patients not pretreated with metformin (Zhang *et al*., 2013). In addition, metformin reduced the proliferation, migration, invasion, sphere formation and stemness characteristics of osteosarcoma cells *in vitro* (Chen *et al*., 2015c). In glioma cells, metformin suppressed spheroid formation and size and inhibited the expression of the glioma stemness‐related marker CD133 (Yuan *et al*., 2018). Although the role of AMPK in the development of cancer stem cells has not been established, there are recent reports demonstrating that HepG2 cells transfected with CD90 have higher expression of CD133 and lower levels of AMPK phosphorylation (Chen *et al*., 2015b), which is in line with our results. Likewise, recent data by Vazquez‐Martin *et al*. demonstrated that AMPK activation by metformin and A‐769662 notably prevented the transcriptional activation of Oct4 and impeded the reprogramming of mouse embryonic fibroblasts (Vazquez‐Martin *et al*., 2012), which is in good agreement with our results. The metformin‐induced attenuation of cancer stem cell features has also been reported by two other groups. Bao *et al*. (2012) demonstrated that metformin significantly decreased cell survival, clonogenicity, sphere‐forming capacity and the expression of Oct4, Nanog and other CSC markers in pancreatic cells. Furthermore, Jung *et al*. (2011) reported that metformin decreased the size and number of mammospheres and Oct4 expression in breast cancer MCF‐7 cells. These data are in accordance with our results and validate the notion that AMPK activation prevents CSC function.

Recent data demonstrated that overexpression of the Wnt/β‐catenin signalling pathway induces rapid embryonic stem cell differentiation (Sun *et al*., 2017). However, the role of Wnt/β‐catenin signalling in stem cell reprogramming is controversial, as it seems that this pathway is involved in both the maintenance of potency and the induction of differentiation (Miki *et al*., 2011). Our data indicate that β‐catenin and cyclin D1 are downregulated in stem‐like cells and that AMPK overexpression increases the levels of both proteins, suggesting a role for this signalling pathway in the induction of HCC stem cell differentiation. The transcriptional coactivator PGC1α is a master regulator of mitochondrial biogenesis and energy expenditure. PGC‐1α directly activates multiple transcription factors to regulate the expression of a plethora of genes. Interestingly, AMPK can regulate PGC‐1α by increasing its expression and phosphorylation. PGC‐1α, in turn, has powerful transcriptional activity by interacting with many different transcription factors, such as PPARγ. Our data reveal that all these proteins are downregulated in sorafenib‐induced stem‐like HCC cells, consistent with their AMPK values and increase in AMPK‐transfected cells. In contrast, the hypoxia‐inducible factor HIF‐1α, which has been revealed as a master regulator of the stemness properties of cancer stem cells (Mimeault and Batra, 2013), is increased in HepG2SF1 and Huh7 cells compared with their parental cells and decreased in AMPK‐transfected cells. These results are in good agreement with previous results suggesting that the reduction in AMPK activity is sufficient to increase HIF‐1α protein levels in cancer cells under normoxic conditions (Faubert *et al*., 2013) and with the notion that HIF‐1α regulates the expression of stem cell markers such as Oct4, Sox2 and Nanog in cancer cells (Lu *et al*., 2015; Zhang *et al*., 2016). Likewise, in hepatospheres obtained from hepatocellular carcinoma cell lines by incubation in a defined medium, a marked increase in ABCB1A and HIF‐1α was observed and was associated with drug resistance (Hashimoto *et al*., 2014), concordant with our results.

## 5 Conclusions

Our data demonstrate that the downregulation of AMPK by sorafenib treatment induces the enrichment of HCC cells in CSCs, which is probably mediated by HIF‐1α. These CSCs have enhanced tumorigenicity, clonogenic ability and differentiation capacity when cultured in specific media as well as enhanced expression of the stem cell markers Oct4, Nanog, ALDH1A1, ABCB1A and AFP. All these features can be reversed by AMPK activation or overexpression. In addition, AMPK upregulation restores sensibility to sorafenib. Our results suggest a novel role for AMPK in the chemotherapy resistance of hepatocellular carcinoma and provide a new therapeutic strategy for HCC.

## Conflict of interest

The authors declare no conflict of interest.

## Author contributions

AB designed and performed the experiments and validated and performed the formal analysis of data. BS designed and performed the experiments. PMG contributed with methodology and critical reading of the manuscript. DVC contributed with resources, methodology and critical reading of the manuscript. NRH contributed with resources, the development of methodology and revision of the manuscript and IDL designed the study, wrote the paper, supervised the research and acquired the financial support. All authors reviewed the manuscript.

## Supporting information


**Fig. S1.** Expression of stem cell markers in hepatocellular carcinoma cells treated with sorafenib for 6 and 12 months. HepG2 and Huh7 cells were cultured continuously for 12 months with a step‐wise increase in sorafenib concentrations (0.75–8 μm). (A) The levels of AFP, CD133 and ALDH1A1 were determined by western blot at 6 (cells grown in 4 μm sorafenib) and 12 (cells grown in 8 μm sorafenib) months. β‐Actin is shown as a loading control. (B) Flow cytometry histograms of CD133 in HepG2 and Huh7 parental cells and in cells treated with sorafenib for 12 months (HepG2SF1 and Huh7SF1). Images are representative of two independent experiments.

**Fig. S2**. Flow cytometry histograms of CD133 in HepG2, Huh7, HepG2SF1 and Huh7SF1 cells transfected with an siRNA selective for AMPK or with a plasmid containing AMPK α1β1γ1. Images are representative of two independent experiments.

**Fig. S3.** Effect of AMPK inhibition on sorafenib sensitivity in stem‐like HCC cells. (A) Effect of the AMPK inhibitor dorsomorphin (Dorso) on HepG2SF1 and Huh7SF1 cell viability inhibition induced by sorafenib. Cells were treated with or without 5 μm dorsomorphin and sorafenib at the indicated concentrations for 24 h. (B) Effect of AMPK knockdown with an siRNA on HepG2SF1 and Huh7SF1 cell viability inhibition induced by sorafenib. Cell viability was determined by the MTT assay and is expressed as the percentage of the control (DMSO treatment). (C) The levels of phosphorylated and total forms of AMPK and ACC in HCC cells were determined by western blot**.** β‐Tubulin (βTub) is shown as a loading control. A representative image of four different experiments is shown. Densitometric values (mean ± SD, *n *=* *3) relative to controls are shown below. *P <* *0.005 significant difference between resistant and control cells and ^#^ *P *<* *0.005 between treated and nontreated cells by two‐way ANOVA and Tukey's multiple comparisons test. Experiments were run in triplicate and carried out at least two times on separate occasions.

**Fig. S4.** Effect of AMPK transfection or activation on sorafenib sensitivity in HepG2 and Huh7 cells. (A) The levels of pAMPK, AMPK, pACC and ACC in HepG2 and Huh7 cells transfected with AMPK α1β1γ1 were determined by western blot. Densitometric values (mean ± SD, *n *=* *4) relative to controls are shown on the right. (B) Effect of the transient expression of AMPK1β1γ1 on cell viability in HepG2 and Huh7 cells treated with increasing concentrations of sorafenib. Cells were treated with sorafenib at the indicated concentrations for 24 h. Cell viability was determined by the MTT assay and is expressed as the percentage of the control (DMSO treatment). (C) Effect of the AMPK activator A‐769662 and capsaicin on HepG2SF1 and Huh7SF1 cell viability following treatment with increasing concentrations of sorafenib. Cells were treated as described above. Experiments were run in triplicate and carried out at least three times on separate occasions. Data are the mean ± SD, *n *=* *3.*P <* *0.005 significant difference between stem‐like and parental cells by two‐way ANOVA and Tukey's multiple comparisons test, #*P *<* *0.005 significant difference between AMPK‐transfected and nontransfected cells (panel A and B) or between control and treated cells (panels C).

**Fig. S5.** Putative mechanism involved in stemness in cells treated long term with sorafenib. In cells sensitive to sorafenib, high levels of AMPK inhibit HIF‐1α while enhancing PGC1α, PPARγ and β‐catenin. In sorafenib‐resistant cells, long‐term treatment with sorafenib induces a depletion of AMPK, releasing HIF‐1α inhibition, which regulates the transcription of stem‐related genes, such as Nanog and Oct4, promoting stemness.

## Data Availability

The datasets generated and/or analysed during the current study are available at https://doi.org/10.17632/ypw4d3j9wb.1.
