# Supplementary figures and images for: Targeting AMP‐activated kinase impacts hepatocellular cancer stem cells induced by long‐term treatment with sorafenib
Source: Mol Oncol. 2019 Apr 15;13(5):1311–31. doi: 10.1002/1878-0261.12488 (PMC6487713; doi:10.1002/1878-0261.12488)

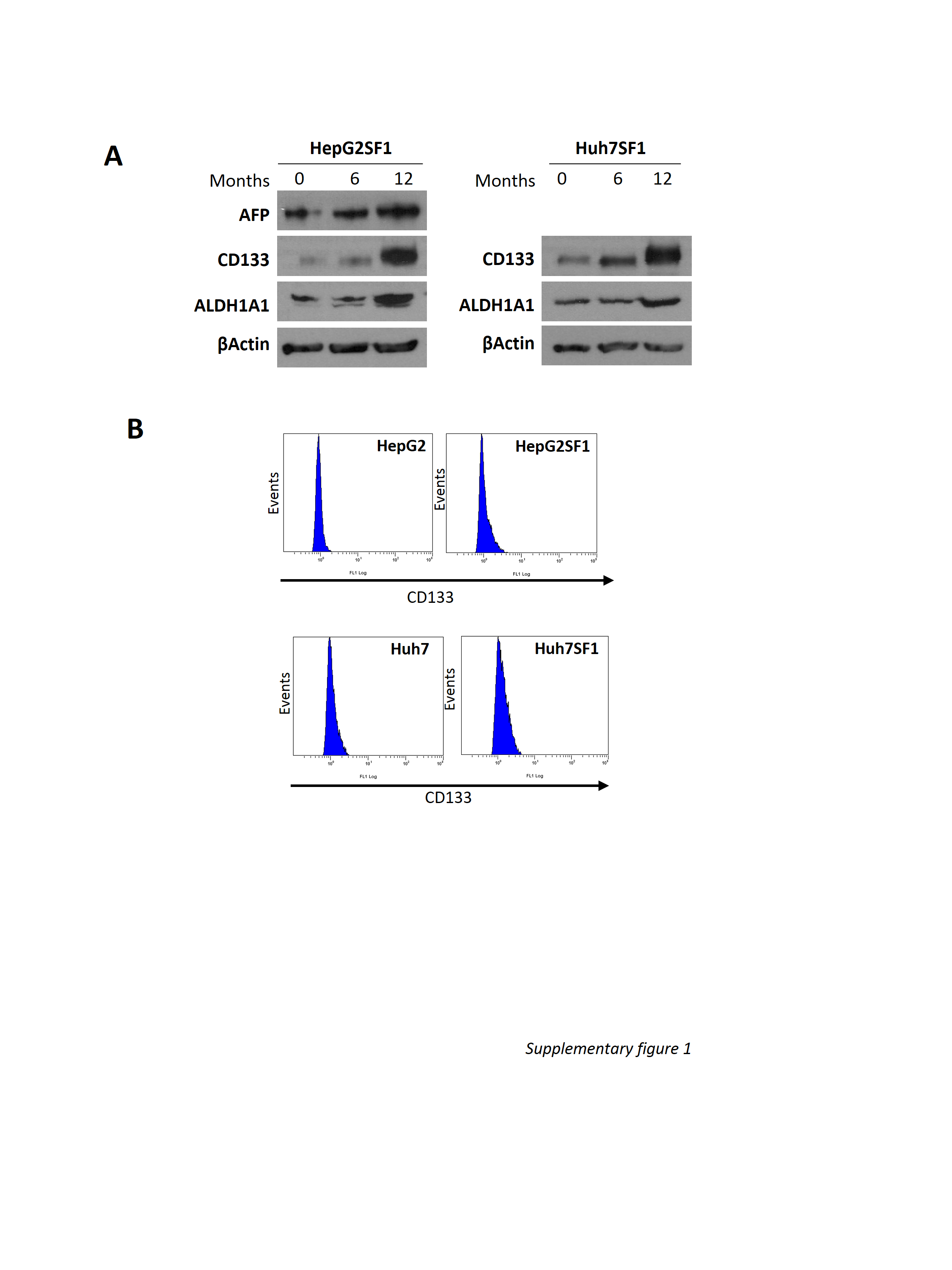

Supplement: Supplementary file 1 — Fig. S1. Expression of stem cell markers in hepatocellular carcinoma cells treated with sorafenib for 6 and 12 months. HepG2 and Huh7 cells were cultured continuously for 12 months with a step‐wise increase in sorafenib concentrations (0.75–8 μm). (A) The levels of AFP, CD133 and ALDH1A1 were determined by western blot at 6 (cells grown in 4 μm sorafenib) and 12 (cells grown in 8 μm sorafenib) months. β‐Actin is shown as a loading control. (B) Flow cytometry histograms of CD133 in HepG2 and Huh7 parental cells and in cells treated with sorafenib for 12 months (HepG2SF1 and Huh7SF1). Images are representative of two independent experiments. [file MOL2-13-1311-s001.tif]

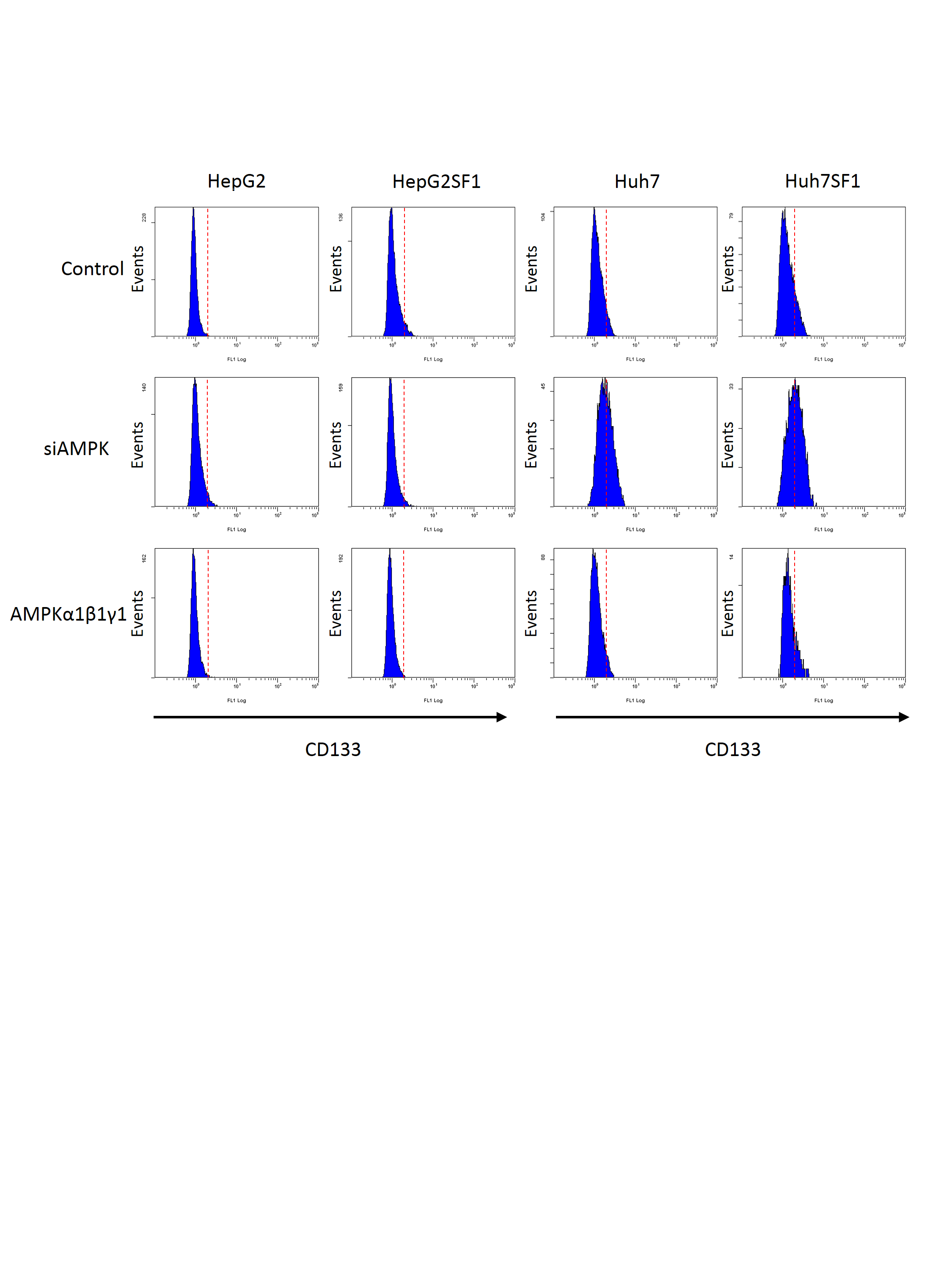

Supplement: Supplementary file 2 — Fig. S2. Flow cytometry histograms of CD133 in HepG2, Huh7, HepG2SF1 and Huh7SF1 cells transfected with an siRNA selective for AMPK or with a plasmid containing AMPK α1β1γ1. Images are representative of two independent experiments. [file MOL2-13-1311-s002.tif]

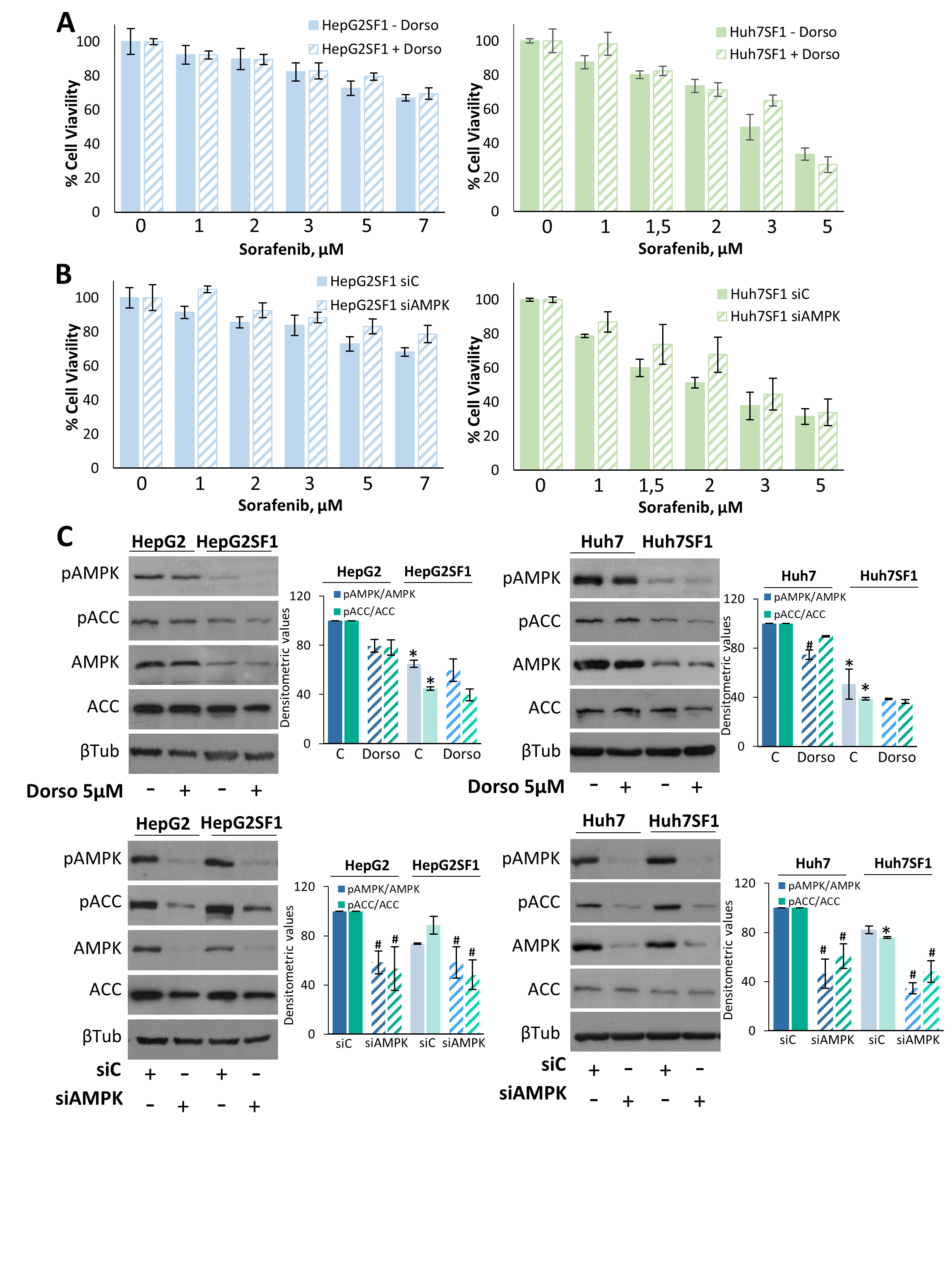

Supplement: Supplementary file 3 — Fig. S3. Effect of AMPK inhibition on sorafenib sensitivity in stem‐like HCC cells. (A) Effect of the AMPK inhibitor dorsomorphin (Dorso) on HepG2SF1 and Huh7SF1 cell viability inhibition induced by sorafenib. Cells were treated with or without 5 μm dorsomorphin and sorafenib at the indicated concentrations for 24 h. (B) Effect of AMPK knockdown with an siRNA on HepG2SF1 and Huh7SF1 cell viability inhibition induced by sorafenib. Cell viability was determined by the MTT assay and is expressed as the percentage of the control (DMSO treatment). (C) The levels of phosphorylated and total forms of AMPK and ACC in HCC cells were determined by western blot. β‐Tubulin (βTub) is shown as a loading control. A representative image of four different experiments is shown. Densitometric values (mean ± SD, n = 3) relative to controls are shown below. *P < 0.005 significant difference between resistant and control cells and # P < 0.005 between treated and nontreated cells by two‐way ANOVA and Tukey's multiple comparisons test. Experiments were run in triplicate and carried out at least two times on separate occasions. [file MOL2-13-1311-s003.tif]

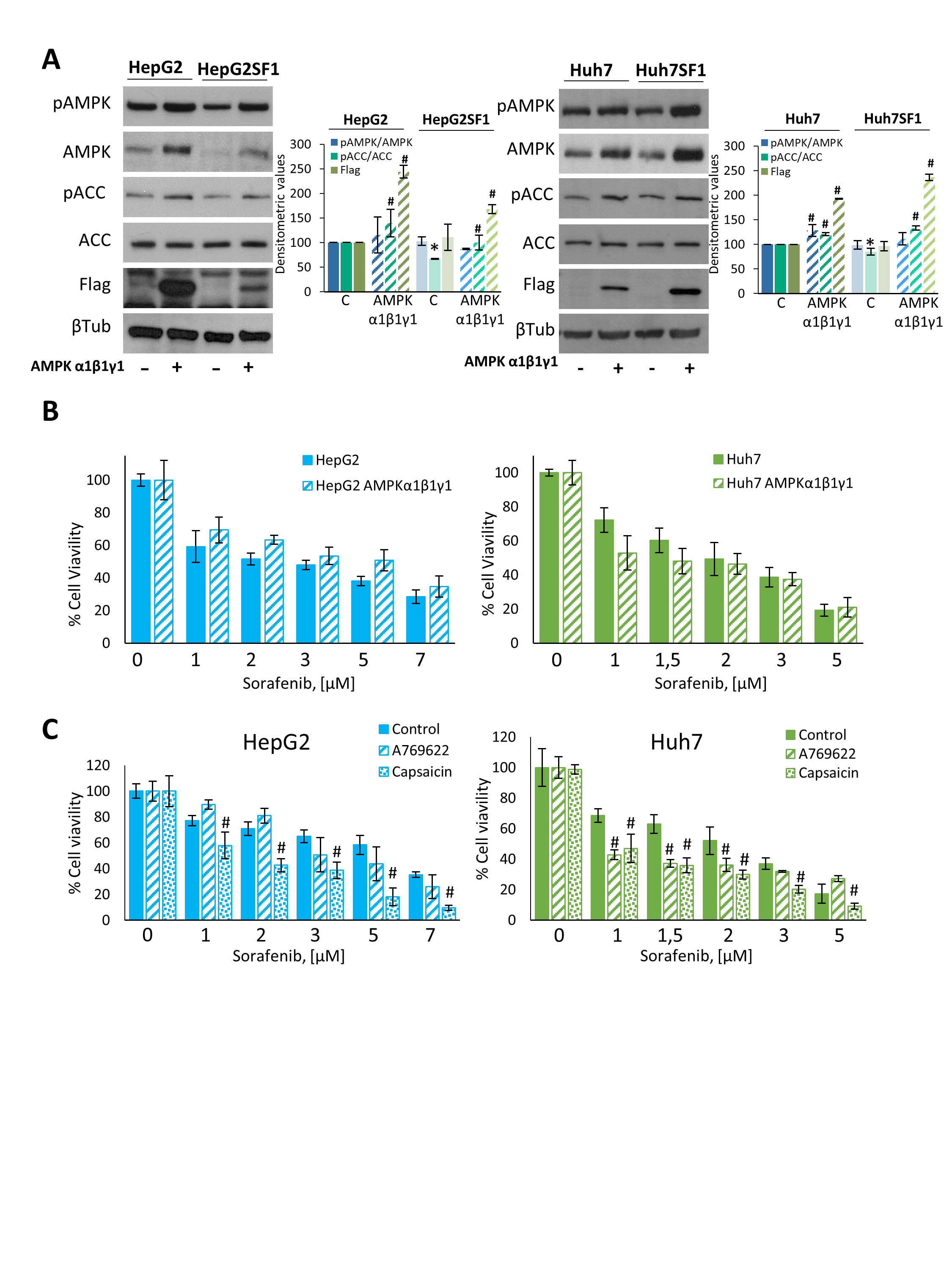

Supplement: Supplementary file 4 — Fig. S4. Effect of AMPK transfection or activation on sorafenib sensitivity in HepG2 and Huh7 cells. (A) The levels of pAMPK, AMPK, pACC and ACC in HepG2 and Huh7 cells transfected with AMPK α1β1γ1 were determined by western blot. Densitometric values (mean ± SD, n = 4) relative to controls are shown on the right. (B) Effect of the transient expression of AMPK1β1γ1 on cell viability in HepG2 and Huh7 cells treated with increasing concentrations of sorafenib. Cells were treated with sorafenib at the indicated concentrations for 24 h. Cell viability was determined by the MTT assay and is expressed as the percentage of the control (DMSO treatment). (C) Effect of the AMPK activator A‐769662 and capsaicin on HepG2SF1 and Huh7SF1 cell viability following treatment with increasing concentrations of sorafenib. Cells were treated as described above. Experiments were run in triplicate and carried out at least three times on separate occasions. Data are the mean ± SD, n = 3.*P < 0.005 significant difference between stem‐like and parental cells by two‐way ANOVA and Tukey's multiple comparisons test, #P < 0.005 significant difference between AMPK‐transfected and nontransfected cells (panel A and B) or between control and treated cells (panels C). [file MOL2-13-1311-s004.tif]

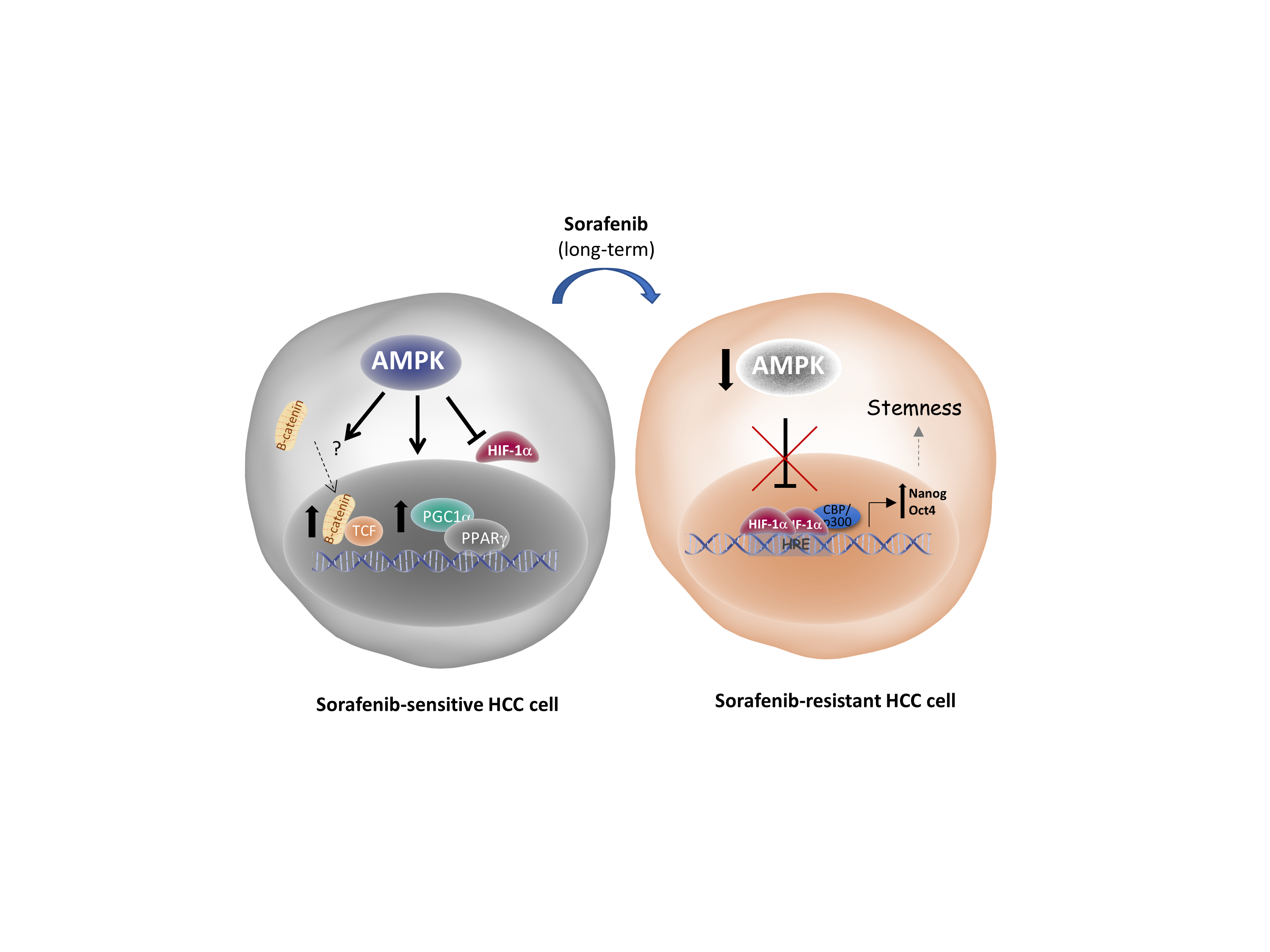

Supplement: Supplementary file 5 — Fig. S5. Putative mechanism involved in stemness in cells treated long term with sorafenib. In cells sensitive to sorafenib, high levels of AMPK inhibit HIF‐1α while enhancing PGC1α, PPARγ and β‐catenin. In sorafenib‐resistant cells, long‐term treatment with sorafenib induces a depletion of AMPK, releasing HIF‐1α inhibition, which regulates the transcription of stem‐related genes, such as Nanog and Oct4, promoting stemness. [file MOL2-13-1311-s005.tif]
